# Plant-based diets, genetic predisposition and risk of non-alcoholic fatty liver disease

**DOI:** 10.1186/s12916-023-03028-w

**Published:** 2023-09-12

**Authors:** Yanling Lv, Shuang Rong, Yan Deng, Wei Bao, Yang Xia, Liangkai Chen

**Affiliations:** 1 Department of Nutrition and Food Hygiene, Hubei Key Laboratory of Food Nutrition and Safety, School of Public Health, Tongji Medical College, Huazhong University of Science and Technology, 13 Hangkong Road, Wuhan, 430030 China; 2 Ministry of Education Key Lab of Environment and Health, School of Public Health, Tongji Medical College, Huazhong University of Science and Technology, Wuhan, 430030 China; 3 Department of Preventive Medicine, School of Public Health, Wuhan University, No.115 Donghu Road, Wuhan, 430071 Hubei China; 4 Department of Nutrition, Hygiene and Toxicology, Academy of Nutrition and Health, School of Public Health, Medical College, Wuhan University of Science and Technology, Wuhan, 430065 China; 5 Division of Life Sciences and Medicine, University of Science and Technology of China, Hefei, 230026 China; 6 Department of Clinical Epidemiology, Shengjing Hospital of China Medical University, Shenyang, China

**Keywords:** Plant-based diet, NAFLD, Genetic risk, UK Biobank

## Abstract

**Background:**

Diets rich in plant-based foods are associated with lower risks of non-alcoholic fatty liver disease (NAFLD), while the prospective evidence is limited. We aimed to examine longitudinal associations of plant-based diets and genetic susceptibility with NAFLD risk.

**Methods:**

This longitudinal cohort study included 159,222 participants (58.0 ± 8.0 years old, 55.7% female) free of NAFLD in the UK Biobank. We calculated the overall plant-based diet index (PDI), the healthful plant-based diet index (hPDI), and the unhealthful plant-based diet index (uPDI). New-onset NAFLD was the primary outcome. The weighted polygenic risk score was calculated based on risk variants associated with NAFLD. Hazard ratios (HR) and 95% confidential intervals (CI) were estimated by Cox proportional hazards model. Magnetic resonance imaging-derived proton density fat fraction (MRI-PDFF) measured liver fat content in a subsample of 20,692 participants (57.5 ± 7.4 years old, 52.6% female) was the secondary outcome. The associations between plant-based diet indices and MRI-PDFF were evaluated using generalized linear models.

**Results:**

During a median follow-up of 9.5 years, 1541 new-onset NAFLD cases were documented. Compared to the lowest quintile, multivariable-adjusted hazard ratios (HRs) of NAFLD in the highest quintile were 0.78 (95% confidential intervals [CI], 0.66–0.93, *p*-trend =0.02), 0.74 (95% CI, 0.62–0.87, *p*-trend <0.0001), and 1.24 (95% CI, 1.05–1.46, *p*-trend = 0.02) for overall PDI, hPDI, and uPDI, respectively. For liver fat content, higher overall PDI and hPDI were associated with lower MRI-PDFF, while higher uPDI was associated with higher liver fat content. We observed a significant interaction between hPDI and PRS (*p*-interaction =0.03), and the NAFLD risk was lowest among participants with the highest hPDI and low genetic risk.

**Conclusions:**

Higher intake of plant-based diets especially healthful plant-based diets was associated with lower NAFLD risk and liver fat content regardless of genetic susceptibility, whereas an unhealthful plant-based diet was associated with higher NAFLD risk and intrahepatic steatosis. These results suggest that the quality of plant-based foods should be highlighted when adopting a plant-based diet.

**Supplementary Information:**

The online version contains supplementary material available at 10.1186/s12916-023-03028-w.

## Background

Non-alcoholic fatty liver disease (NAFLD) has emerged as the most common chronic liver disorder globally [1]. Currently, nearly 32.4% of adults worldwide have NAFLD [2], while around 1 in 3 people has an early stage of the disease in the UK [3]. Diet is an essential modifiable risk factor for NAFLD, and dietary patterns, in which nutrients and foods are consumed in combination, reflect real-world dietary practice [4].

Plant-based diet patterns nowadays are gaining attention as their environmental sustainability benefits [5], and diet patterns characterized by high consumption of healthy plant-based foods were associated with lower NAFLD prevalence and liver fat content [6, 7]. However, not all plant-based foods were beneficial to NAFLD, as less nutrient-dense plant foods, including refined grains, fruit juices, and sugar-sweetened beverages, are associated with higher NAFLD risk [8–10]. To distinguish the plant-based diets with different quality, previous studies have developed three plant-based diet indices (PDIs), an overall plant-based diet index (PDI), which emphasizes the intake of all plant foods; a healthful plant-based diet index (hPDI), which emphasizes higher consumption of healthful plant-based foods such as whole grains, vegetables, nuts, legumes, coffee, and tea; and an unhealthful plant-based index (uPDI), which highlights the consumption of less healthful plant-based foods associated with increased risks of several chronic diseases [11]. The associations of lower overall PDI and hPDI but higher uPDI with higher liver fat content and the prevalence of fatty liver have been reported [12, 13], while insignificant associations were shown in other studies [14, 15]. Given the limited sample size and inconsistent findings of existing studies, evidence from large population-based studies with a prospective design is warranted.

The development of NAFLD is a consequence of an interaction between environmental and genetic factors [16]. To date, several NAFLD-associated loci have been identified in genome-wide association studies [17]. However, no studies have examined the interaction between diet patterns and genetic predisposition on NAFLD primary prevention, and only one study showed that improved adherence to the Mediterranean diet pattern or the Alternative Healthy Eating Index (AHEI) was associated with reduced liver fat content, particularly among individuals with high genetic risk [18]. Therefore, our study aimed to longitudinally investigate the association between PDIs and NAFLD risk and to explore whether such associations would be modified by the genetic risk of NAFLD.

## Methods

### Study design and setting

UK Biobank recruited more than 0.5 million participants aged 37-73 years from the general population between 2006 and 2010. Participants attended one of 22 assessment centers across England, Scotland, and Wales, where they completed the touch-screen questionnaire, a face-to-face interview with a nurse, a series of physical measurements, and provided biological samples. The date and cause of hospital admissions were obtained through recorded linkage to health episode statistics (England and Wales) and Scottish morbidity records (Scotland). The UK Biobank study was approved by the North West Multi-centre Research Ethics Committee (REC reference for UK Biobank 11/NW/0382), and written informed consent was provided prior to participation. Data from the UK Biobank are available to all researchers upon application (https://www.ukbiobank.ac.uk/).

### Study population

We included participants with at least one dietary assessment (*n* = 210,673) and excluded those with diagnosed NAFLD, cirrhosis, or other liver diseases when dietary information collection was completed (*n* = 2920). We further excluded participants diagnosed with alcohol-related diseases at the end of the follow-up (*n* = 266). Participants with implausible energy intake (< 800 or > 4200 kcal/day in males and < 600 or > 3500 kcal/day in females) (*n* = 2994), without complete genetic data, or not of European descent were also excluded (*n* = 13,111). We excluded participants with cardiovascular diseases or cancer at baseline as they likely changed eating habits after disease diagnosis (*n* = 32,160). Finally, 159,222 participants were included in the NAFLD risk analyses and 20,692 in liver fat content analyses (Additional file 1: Figure S1).

### Dietary assessment

Dietary information in UK Biobank was collected using the Oxford WebQ based on a 24-h dietary recall questionnaire. The Oxford WebQ has been validated against an interviewer-administered 24-h recall [19] and biomarkers [20]. The consumption of more than 200 commonly consumed food and more than 30 beverage items over the previous 24 h were collected. The first instance of dietary assessment was conducted in the assessment centers for the last 70,000 participants from April 2009 to September 2010, and the following 4 online cycles were conducted through e-mail invitations on four separate occasions between February 2011 and April 2012. For those who completed twice and more, the intake of every food item was calculated as the means of intake answered in all diet assessments.

We calculated the overall PDI, hPDI, and uPDI using the established method conceptualized by Satija et al. [11, 21]. We categorized foods into 17 groups (whole grains, fruits, vegetables, nuts, legumes, tea and coffee, refined grains, potatoes, sugary drinks, fruit juices, sweets and desserts, animal fat, dairy, eggs, fish or seafood, meat, and miscellaneous animal-based foods) and classed them into larger categories: healthy plant-based foods, less healthy plant-based foods, and animal foods (Additional file 1: Table S1). Given the controversial health effect of alcohol, we did not include alcoholic beverages in plant-based indices but adjusted them in the regression model [22]. The intakes of every food group were ranked into quintiles and given positive (Q1 to Q5 received 1 to 5 point) or reverse (Q1 to Q5 received 5 to 1 point) scores. To generate the overall PDI, healthful and unhealthful plant-based food groups were given positive scores, and animal food groups received reverse scores. For hPDI, positive scores were given to healthy plant-based food groups, and reverse scores were given to less healthy plant-based food groups and animal food groups. Finally, for creating uPDI, positive scores were given to less healthy plant-based food groups, and reverse scores were given to healthy plant-based food groups and animal food groups. The scores of 17 food groups for an individual were summed up to obtain the PDIs, with a theoretical range from 17 to 85.

### Ascertainment of NAFLD

NAFLD was diagnosed according to hospital inpatient records, death registry data, and primary care data linked to the UK Biobank based on the 9th and 10th International Classification of Diseases (ICD-9 and ICD-10) [23]. The detailed ICD and primary care read codes are shown in Additional file 1: Table S2. The time-to-event was calculated from the last dietary assessment to the date of NAFLD diagnosis, death, loss to follow-up, or censorship (30 September 2021 for England, 31 July 2021 for Scotland, and 28 February 2018 for Wales), whichever came first.

### Magnetic resonance imaging (MRI) scan of the liver

The MRI imaging protocol and analysis of liver fat content in the UK Biobank have been previously published [24]. Briefly, the liver MRI scan was performed using Siemens 1.5T MAGNETOM Aera by LiverMultiScan©, which is part of the abdominal imaging protocol in the UK Biobank. MRI-derived proton density fat fraction (MRI-PDFF), which has the highest accuracy for quantification of intrahepatic fat content compared with other non-invasive imaging modalities and positively correlates with histopathological hepatic triglyceride content [25, 26], was derived as previously described [24, 27]. Fat-referenced PDFF was measured as the average PDFF of nine regions of interest in the liver, placed while avoiding any inhomogeneities, major vessels, or bile ducts.

### Polygenic risk score (PRS) for NAFLD

The detail of genotyping, imputation, and quality control of genetic data in UK Biobank has been discussed elsewhere [28]. We calculated the PRS of NAFLD based on 5 single-nucleotide polymorphisms (SNPs) significantly associated with NAFLD in participants of European descent (rs738409, rs58542926, rs641738, rs1260326, and rs72613567) [17]. The effect size of each SNP (β-coefficient) and other related information are shown in Additional file 1: Table S3. The PRS of NAFLD was calculated by summing the risk allele numbers of each SNP weighted by the effect size to NAFLD: PRS = β1 × SNP1 + β2 × SNP2 + …+βn × SNPn, where SNPn is the risk allele number of each SNP.

### Covariates

Age at dietary assessment was determined from the date of birth to the date completed the last dietary assessment. Sex (male or female), education (lower secondary, upper secondary, vocational, college or university, or others), and household income (< 18,000, 18,000–30,999, 31,000–51,999, 52,000–100,000, > £100,000 £/year) were self-reported. Socioeconomic status was reflected by the Townsend deprivation index (quintiles) derived from the postcode of residence [29]. Smoking status was defined as current, former, or never. Physical activity was estimated in metabolic equivalent minutes per week (MET-mins/week, categorized into < 600, 600–1199, ≥ 1200 MET-mins/week, or unknown). Body mass index was calculated as weight in kilograms divided by height in meters squared (< 25.0, 25.0–29.9, ≥ 30 kg/m^2^, or unknown). Alcohol consumption (0, 0.1–10.0, 10.1–20.0, 20.1–35.0, ≥ 35.1 g/day, or unknown) and total energy intake (in Kcal, quintiles) were estimated using 24-h dietary recall data.

### Statistical analysis

The analysis plan was preregistered with the Open Science Foundation (https://osf.io/z9u5m/). All analyses were conducted using SAS 9.4 (SAS Institute Inc) and R software (version 4.2.1). All statistical tests were 2-tailed, and *p* < 0.05 was considered statistically significant.

Associations between PDIs and NAFLD were estimated using Cox proportional hazard regression model by quintiles of exposures with time-to-event as the timescale. The results were presented as hazard ratios (HRs) and 95% confidence intervals (CIs). The proportional hazards assumption was tested by the Schoenfeld residual method and satisfied. The potential confounders were adjusted based on a priori-defined directed acyclic graph (Additional file 1: Figure S2). The minimize model was adjusted for age and sex. The multivariable-adjusted model was further adjusted for education, household income, Townsend deprivation index, assessment centers, smoking status, alcohol consumption, physical activity, total energy intake, BMI, NAFLD-PRS, the first 10 principal components of ancestry, and the genotype measurement batch. Given that BMI could be on the causal pathway between PDIs and NAFLD risk, we additionally conducted a multivariable-adjusted model without BMI. PDIs were also treated as continuous variables, and HR per 10-point increment was reported. *P* for trend was estimated by including a continuous variable assigned each quintile to its median value. The cumulative risk curves of NAFLD by PDIs quintiles were plotted using Kaplan–Meier methods. To investigate the dose-response associations between PDIs and NAFLD risk, we performed restricted cubic spline regressions (RCS) fitted by Cox hazard regression with four knots (5th, 35th, 65th, and 95th) to flexibly model the NAFLD risk distributed by PDIs (trimmed with 2.5% and 97.5% of the distribution). Furthermore, to explore the associations of PDIs with intrahepatic fat content, we estimated *β*-coefficients (95% CI) of PDIs quintiles with MRI-PDFF by generalized linear models and modeled the curves of continuous PDIs and MRI-PDFF using generalized additive models.

We conducted stratified analysis by NAFLD genetic risk tertiles and multiplicative interactions were tested by including a PDIs×PRS term in the fully adjusted model. We further conducted the sex-specific interaction analyses between PRS and PDIs on NAFLD risk to determine whether the interactions would differ by sex. We also estimated the joint association of PDIs and genetic risk with NAFLD risk and MRI-PDFF by defining a combined variable based on tertiles of genetic risk and PDIs (9 categories) with the highest risk combination (the lowest overall PDI/hPDI and the highest PRS) or the lowest (the lowest uPDI and the lowest PRS) as reference.

As secondary analyses, we (1) used sex-specific quintiles of PDIs and reran the main analysis; (2) conducted the stratified analyses of NAFLD risk and MRI-PDFF by age, sex, obesity, energy intake, alcohol consumption, and physical activity; (3) estimated the mediating effect of BMI at the second assessment on the associations between PDIs and NAFLD risk; (4) individually excluded each of 17 food groups from the PDIs and assessed the associations between those modified indices and NAFLD risk with further adjusting for the intake of excluded food group; (5) further adjusted for diagnosed depression, dyslipidemia, hypertension, and diabetes at baseline (i.e., the date when the last dietary assessment was completed) to limit the potential confounding of chronic disorders; (6) further adjusted for liver function to control the confounding effect of baseline liver function; (7) further adjusted for glucose, glycated hemoglobin, and triglyceride to minimize the confounding of metabolic factors; (8) further adjusted for waist circumference to limit the confounding of central obesity; (9) excluded participants with less than twice dietary assessment; and (10) excluded participants with less than 2 years of follow-up to minimize the reverse casualty.

## Results

The baseline characteristics of 159,222 participants are shown in Table 1. The mean age was 58.0 ± 8.0, and 55.7% were female. The mean (SD) times of 24-h dietary assessment were 2.2 (1.2). The overall PDI ranged from 25 to 74, hPDI ranged from 27 to 82, and uPDI ranged from 27 to 78. Participants with higher overall PDI and hPDI but lower uPDI tended to be female, well-educated, non-current smokers, and with lower BMI. Total energy intake was higher among participants with higher overall PDI but lower in those with higher hPDI and uPDI. The baseline characteristics and PDIs were generally consistent among total participants and those with MRI-PDFF data (*n* = 20,692, Additional file 1: Table S4). The baseline characteristics by NAFLD status are shown in Additional file 1: Table S5.

**Table 1:** Baseline characteristics of 159,222 participants by three plant-based diet indices

Characteristics	**Overall PDI**		**hPDI**		**uPDI**	
	Quintile 1 (*n* = 29,542)	Quintile 5 (*n* = 28,808)	Quintile 1 (*n* = 33,921)	Quintile 5 (*n* = 31,175)	Quintile 1 (*n* = 32,351)	Quintile 5 (*n* = 31,201)
Age, mean (SD), years	57.2 (8.0)	58.2 (7.9)	56.4 (8.3)	58.7 (7.6)	59.6 (7.4)	55.5 (8.1)
Male, *n* (%)	15,980 (54.1)	10,909 (37.9)	19,288 (56.9)	10,162 (32.6)	13,762 (42.5)	14,760 (47.3)
Townsend Deprivation Index, median (IQR)	− 2.2 (− 3.6, 0.3)	− 2.5 (− 3.8, − 0.3)	− 2.3 (− 3.7, − 0.05)	− 2.3 (− 3.7, − 0.03)	− 2.4 (− 3.8, − 0.2)	− 2.3 (− 3.7, 0.1)
Deprivation fifth, *n* (%)						
First (least deprived)	5321 (18.0)	5908 (20.5)	6611 (19.5)	6061 (19.4)	6562 (20.3)	5988 (19.2)
Second to fourth	17,273 (58.5)	17,412 (60.5)	20,266 (59.8)	18,650 (59.8)	19,462 (60.2)	18,387 (58.9)
Fifth (most deprived)	6902 (23.4)	5460 (19.0)	6992 (20.6)	6435 (20.6)	6293 (19.5)	6775 (21.7)
Unknown	46 (0.2)	28 (0.1)	52 (0.2)	29 (0.1)	34 (0.1)	51 (0.2)
Education						
College or university	11,127 (37.7)	13,912 (48.3)	13,376 (39.4)	14,926 (47.9)	15,380 (47.5)	11,831 (37.9)
Vocational	3199 (10.8)	2760 (9.6)	3472 (10.2)	3098 (9.9)	3213 (9.9)	3087 (9.9)
Upper secondary	3893 (13.2)	3952 (13.7)	4816 (14.2)	4026 (12.9)	4228 (13.1)	4400 (14.1)
Lower secondary	8249 (27.9)	6342 (22.0)	9528 (28.1)	6823 (21.9)	7096 (21.9)	9241 (29.6)
Others	2938 (10.0)	1765 (6.1)	2597 (7.7)	2190 (7.0)	2342 (7.2)	2498 (8.0)
Unknown	136 (0.5)	77 (0.3)	132 (0.4)	112 (0.4)	92 (0.3)	144 (0.5)
Household income, £						
< 18,000	3944 (13.4)	3705 (12.9)	4160 (12.3)	4133 (13.3)	4430 (13.7)	3814 (12.2)
18,000–30,999	5883 (19.9)	6261 (21.7)	6927 (20.4)	6613 (21.2)	7297 (22.6)	6188 (19.8)
31,000–51,999	7736 (26.2)	7613 (26.4)	9186 (27.1)	7957 (25.5)	8306 (25.7)	8269 (26.5)
52,000–100,000	6865 (23.2)	6667 (23.1)	8251 (24.3)	7104 (22.8)	6977 (21.6)	7599 (24.4)
> 100,000	2231 (7.6)	1800 (6.3)	2424 (7.2)	2161 (6.9)	2199 (6.8)	2287 (7.3)
Unknown	2883 (9.8)	2762 (9.6)	2973 (8.8)	3207 (10.3)	3142 (9.7)	3044 (9.8)
Alcohol consumption among drinkers, median (IQR), g/day	14.5 (6.2, 27.4)	10.2 (3.6, 18.2)	12.8 (5.1, 24.1)	10.2 (4.3, 19.9)	11.2 (5.1, 20.6)	11.2 (4.5, 22.7)
Current smoker, *n* (%)	3216 (10.9)	1579 (5.5)	3102 (9.1)	1934 (6.2)	2081 (6.4)	2914 (9.3)
Body mass index, mean (SD), kg/m^2^	27.6 (4.8)	26.2 (4.4)	27.7 (4.9)	26.0 (4.3)	26.5 (4.5)	27.3 (4.8)
Waist circumference, mean (SD), cm	91.5 (13.4)	86.4 (12.6)	92.3 (13.4)	85.2 (12.6)	87.7 (13.0)	90.0 (13.4)
Total physical activity, MET-mins/week						
0–599	5366 (18.2)	3444 (12.0)	6259 (18.5)	3626 (11.6)	3966 (12.3)	5785 (18.5)
600–1199	4647 (15.7)	4443 (15.4)	5520 (16.3)	4598 (14.8)	4733 (14.6)	5103 (16.4)
≥ 1200	14,958 (50.6)	16,992 (59.0)	17,133 (50.5)	18,346 (58.9)	19,036 (58.8)	15,430 (49.5)
Unknown	4571 (15.5)	3929 (13.6)	5009 (14.8)	4605 (14.8)	4616 (14.3)	4883 (15.7)
Energy intake (kcal/d)	1917.4 (1560.6, 2314.9)	2147.2 (1836.6, 2510.8)	2273.9 (1943.0, 2652.6)	1791.8 (1500.9, 2128.2)	2113.3 (1783.1, 2492.8)	1922.3 (1589.0, 2287.2)
Alanine aminotransferase, median (IQR), U/L	21.0 (15.9, 28.9)	18.7 (14.6, 24.8)	21.1 (15.9, 29.1)	18.4 (14.5, 24.3)	19.5 (15.2, 25.9)	19.8 (15.0, 27.6)
Albumin, median (IQR), g/L	45.5 (43.7, 47.2)	45.4 (43.7, 47.1)	45.4 (43.7, 47.2)	45.5 (43.8, 47.2)	45.4 (43.7, 47.1)	45.4 (43.7, 47.2)
Gamma glutamyltransferase, median (IQR), U/L	27.7 (19.2, 43.3)	22.2 (16.4, 33.3)	27.3 (19.1, 42.3)	21.9 (16.3, 32.6)	23.6 (17.2, 35.4)	25.5 (17.9, 39.7)
Glucose, mean (SD), mmol/L	5.1 (1.1)	5.0 (1.0)	5.1 (4.9)	5.0 (1.0)	5.1 (1.1)	5.1 (1.1)
Glycated hemoglobin, mean (SD), mmol/mol	35.4 (5.9)	35.1 (5.3)	35.4 (6.2)	35.0 (5.1)	35.5 (5.7)	35.0 (5.6)
Triglycerides, median (IQR), mmol/L	132.4 (93.0, 194.1)	121.6 (86.7, 173.4)	137.7 (96.5, 200.8)	114.3 (83.1, 163.6)	120.4 (86.5, 173.5)	131.4 (92.4, 191.3)
Depression, *n* (%)	4033 (13.7)	4016 (13.9)	4607 (13.6)	4413 (14.2)	4431 (13.7)	4609 (14.8)
Dyslipidemia, *n* (%)	15,007 (50.8)	12,701 (44.1)	17,977 (53.0)	12,755 (40.9)	14,427 (44.6)	15,619 (50.1)
Hypertension, *n* (%)	15,706 (53.2)	13,572 (47.1)	17,502 (51.6)	14,584 (46.8)	16,586 (51.3)	14,981 (48.0)
Diabetes, *n* (%)	1411 (4.8)	965 (3.4)	1614 (4.8)	1106 (3.6)	1488 (4.6)	1205 (3.9)

*Abbreviations*: *hPDI* Healthful plant-based diet index, *IQR* Interquartile range, *PDI* Plant-based diet index, *SD* Standard deviation, *uPDI* Unhealthful plant-based diet index

**Fig. 1:**
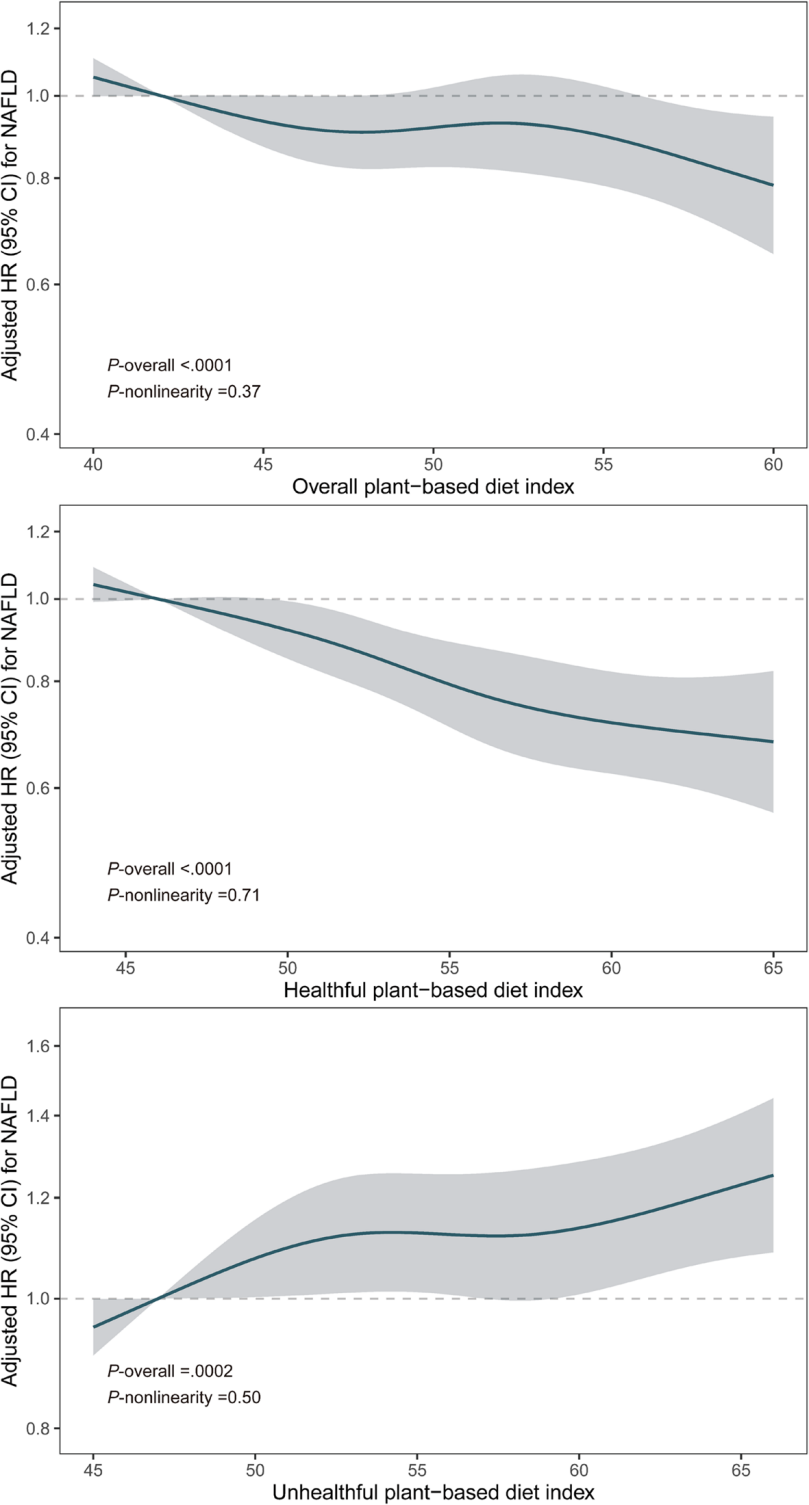
Restrict cubic spline for associations of overall plant-based diet index, healthful plant-based diet index, and unhealthful plant-based diet index with NAFLD risk. Adjusted for age at the last dietary assessment, sex, education, household income, Townsend deprivation index, assessment centers, smoking, alcohol consumption, physical activity, total energy, BMI, NAFLD-PRS, first 10 principal components of ancestry, and genotype measurement batch. Abbreviations: BMI, body mass index; CI, confidence interval; HR, hazards ratio; NAFLD, non-alcoholic fatty liver disease; PRS, polygenic risk score

**Fig. 2:**
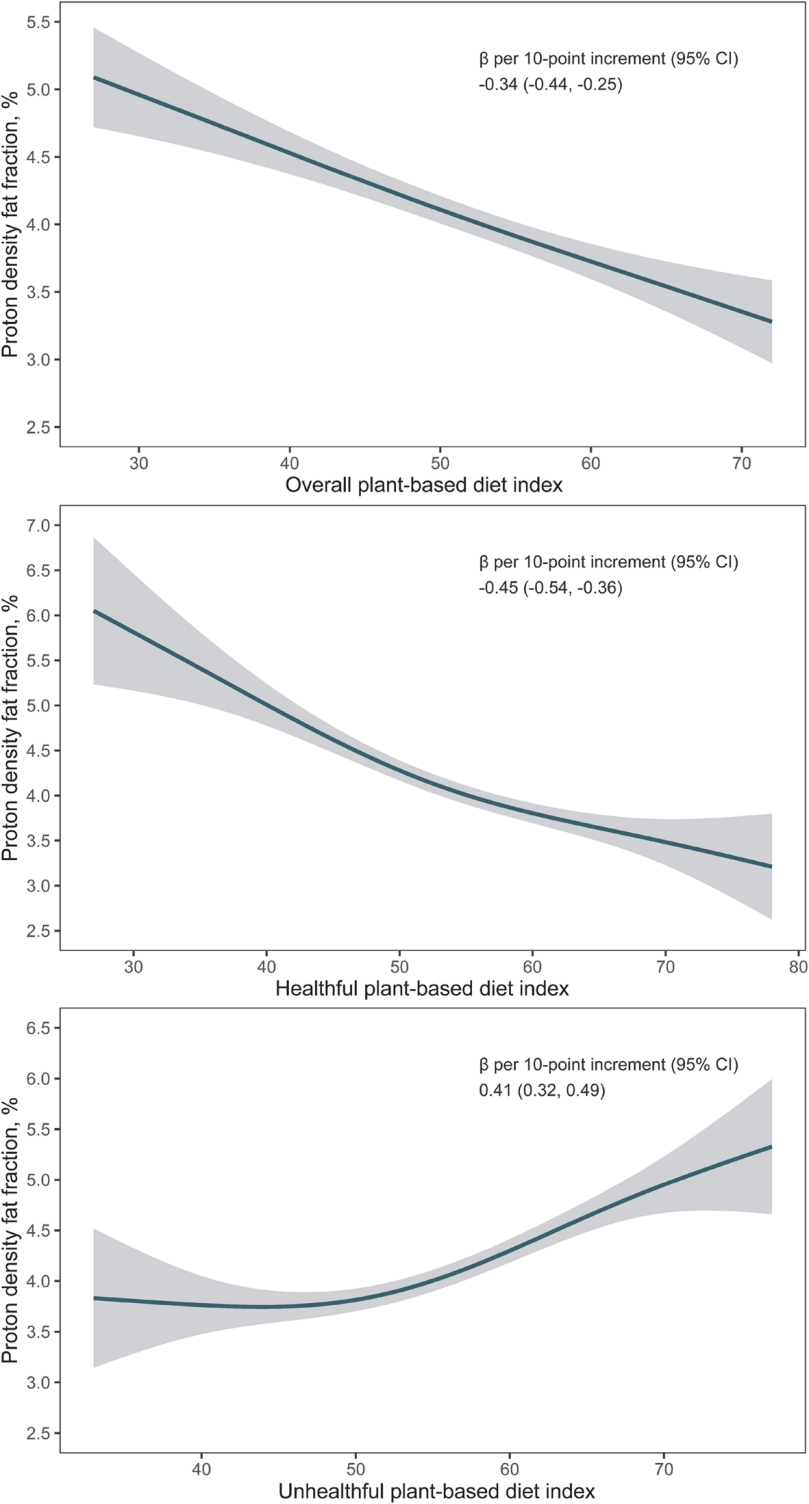
Associations of overall plant-based diet index, healthful plant-based diet index, and unhealthful plant-based diet index with MRI-PDFF. Adjusted for age at the last dietary assessment, age at MRI scan, sex, education, household income, Townsend deprivation index, assessment centers, smoking, alcohol consumption, physical activity, total energy, BMI, NAFLD-PRS, first 10 principal components of ancestry, and genotype measurement batch. Abbreviations: MRI, magnetic resonance imaging; NAFLD, non-alcoholic fatty liver disease; PDFF, proton density fat fraction; PRS, polygenic risk score

During a median follow-up of 9.5 years, 1541 NAFLD cases were documented. We did not observe significant departures from linearity when the non-linearity of overall PDI, hPDI, and uPDI with NAFLD risk was tested using RCS (Fig. 1, *p-*nonlinearity > 0.05 for all PDIs). The cumulative risks of NAFLD by PDIs quintiles are shown in Additional file 1: Figure S3. Compared to the lowest quintile, multivariable-adjusted HRs of NAFLD in the highest quintile were 0.78 (95% CI, 0.66–0.93, *p*-trend = 0.02), 0.74 (95% CI, 0.62–0.87, *p*-trend < 0.0001), and 1.24 (95% CI, 1.05–1.46, *p*-trend = 0.02) for PDI, hPDI, and uPDI, respectively. These associations were stronger when not adjusting for BMI (Table 2). Additionally, per 10-point increment of PDIs was associated with an 11% lower, 20% lower, and 14% higher risk of NAFLD (with HRs 0.89 [95% CI, 0.81–0.97], 0.80 [95% CI, 0.73–0.88], and 1.14 [95% CI, 1.05–1.24] for overall PDI, hPDI, and uPDI, respectively (Table 2). When the liver fat content was indicated by MRI-PDFF and further adjusted for age at MRI in the final model, higher overall PDI and hPDI were associated with lower intrahepatic fat content (*β* [95% CI] per 10-point increment were − 0.34 [− 0.44, − 0.25] and − 0.45 [− 0.54, − 0.36], respectively), while higher uPDI was associated with higher liver fat content (*β* [95% CI], 0.41 [0.32, 0.49], Fig. 2, Additional file 1: Table S6).

**Table 2:** Hazard ratios (95% confidence intervals) of NAFLD according to quintiles of overall plant-based diet index, healthful plant-based diet index, and unhealthful plant-based diet index

	Quintile of dietary score					*P* for trend	Per 10-point increment
	Quintile 1	Quintile 2	Quintile 3	Quintile 4	Quintile 5		
**Overall plant-based diet index**							
Median score	43 (41, 44)	48 (47, 49)	50 (50, 51)	53 (52, 54)	58 (57, 60)		
Cases/person-years	373/291,207	394/375,734	204/222,454	364/381,403	206/279,141		
Age and sex-adjusted model	1.00 (ref.)	**0.83 (0.72, 0.96)**	**0.73 (0.62, 0.87)**	**0.76 (0.66, 0.88)**	**0.59 (0.50, 0.70)**	< .0001	**0.74 (0.68, 0.81)**
Multivariable-adjusted model	1.00 (ref.)	0.94 (0.81, 1.08)	0.87 (0.73, 1.03)	0.96 (0.83, 1.12)	**0.78 (0.66, 0.93)**	0.02	**0.89 (0.81, 0.97)**
Multivariable-adjusted without BMI	1.00 (ref.)	0.89 (0.78, 1.03)	**0.81 (0.68, 0.96)**	**0.86 (0.74, 1.000)**	**0.68 (0.57, 0.81)**	< .0001	**0.81 (0.74, 0.89)**
**Healthful plant-based diet index**							
Median score	47 (45, 48)	51 (50, 52)	54 (53, 55)	57 (56, 58)	62 (61, 64)		
Cases/person-years	448/327,940	297/262,363	278/304,869	282/348,301	236/306,467		
Age and sex-adjusted model	1.00 (ref.)	**0.83 (0.72, 0.96)**	**0.67 (0.58, 0.78)**	**0.60 (0.51, 0.69)**	**0.57 (0.48, 0.67)**	< .0001	**0.68 (0.63, 0.74)**
Multivariable-adjusted model	1.00 (ref.)	0.92 (0.79, 1.06)	**0.78 (0.67, 0.91)**	**0.72 (0.62, 0.85)**	**0.74 (0.62, 0.87)**	< .0001	**0.80 (0.73, 0.88)**
Multivariable-adjusted without BMI	1.00 (ref.)	**0.85 (0.73, 0.98)**	**0.69 (0.59, 0.80)**	**0.62 (0.53, 0.72)**	**0.59 (0.50, 0.70)**	< .0001	**0.70 (0.64, 0.76)**
**Unhealthful plant-based diet index**							
Median score	48 (46, 49)	52 (51, 53)	55 (54, 56)	58 (57, 59)	63 (62, 65)		
Cases/person-years	261/313,738	237/261,857	316/307,456	357/360,255	370/306,634		
Age and sex-adjusted model	1.00 (ref.)	1.09 (0.92, 1.30)	**1.24 (1.05, 1.46)**	**1.20 (1.02, 1.41)**	**1.46 (1.24, 1.72)**	< .0001	**1.27 (1.16, 1.38)**
Multivariable-adjusted model	1.00 (ref.)	1.08 (0.91, 1.29)	**1.21 (1.03, 1.43)**	1.10 (0.94, 1.30)	**1.24 (1.05, 1.46)**	0.02	**1.14 (1.05, 1.24)**
Multivariable-adjusted without BMI	1.00 (ref.)	1.09 (0.92, 1.30)	**1.23 (1.04, 1.45)**	1.16 (0.98, 1.36)	**1.34 (1.14, 1.58)**	0.0006	**1.20 (1.10, 1.31)**

Multivariable-adjusted model further adjusted for education, household income, Townsend deprivation index, assessment centers, smoking, alcohol consumption, physical activity, total energy, BMI, NAFLD-PRS, first 10 principal components of ancestry, and genotype measurement batch. We conducted an additional model without BMI as a covariate given that BMI might be in the PDI and NAFLD pathway

*Abbreviations*: *BMI* Body mass index, *NAFL* Non-alcoholic fatty liver disease, *PRS* Polygenic risk score, *ref*. Reference

When assessing the joint association of PDIs and PRS with NAFLD risk, compared with the highest risk combinations, most other groups for overall PDI and hPDI had significantly lower NAFLD risk, and NAFLD risk was lowest in participants with low genetic risk and highest PDI/hPDI scores. On the contrary, compared to those with the lowest PRS and lowest uPDI, the NAFLD risk was increased with higher PRS and uPDI (Additional file 1: Figure S4). In joint associations of MRI-PDFF, the *β*-coefficients were gradually increased with increased PDIs and PRS (Additional file 1: Figure S5). In stratified analyses by genetic risk, the associations of higher overall PDI and lower uPDI with lower NAFLD risk were not modified by genetic susceptibility to NAFLD, but the association of hPDI with NAFLD risk was significantly modified by genetic risk (*P*-interaction > 0.05 for overall PDI and uPDI,* P*-interaction = 0.03 for hPDI, Additional file 1: Tables S7-S9).

Associations of PDIs with NAFLD risk and liver fat content persisted when using sex-specific quintiles of PDIs, and no significant interaction between sex and PDIs was observed (Additional file 1: Tables S10-S11, Figure S6). In addition, we observed no significant modifications by other major risk factors (age, obesity, energy intake, alcohol consumption, or physical activity) of NAFLD risk or MRI-PDFF, except the association between uPDI and NAFLD risk was modified by obesity (*p* for interaction = 0.0009 < 0.003 (0.05/(3 exposures * 5 groups)), Additional file 1: Tables S12-S13). Associations between PDIs and NAFLD remained largely unchanged when we further adjusted for chronic disorders and liver functions (Additional file 1: Table S14, sensitivity analysis 1&2); when we further adjusted for metabolic indicators and waist circumference (Additional file 1: Table S14, sensitivity analysis 3&4); when we excluded participants with less than twice diet assessments (Additional file 1: Table S14, sensitivity analysis 5); and when we excluded participants with less than 2 years of follow-up (Additional file 1: Table S14, sensitivity analysis 6). In addition, we observed significant mediating effects of BMI on PDIs-NAFLD risk associations, which were 51.8%, 47.0%, and 46.5% for PDI, hPDI, and uPDI, respectively (Additional file 1: Table S15). Furthermore, when we excluded each one of 17 food groups at a time from PDIs and adjusted for the excluded food group intake, the adjusted HRs for each 10-point increment in PDIs were not substantially altered. However, higher intakes of nuts, tea, and coffee were associated with lower NAFLD risk, but higher intakes of sugar-sweetened beverages, fish, and sea foods were associated with higher NAFLD risk (Additional file 1: Table S16). These results indicated that associations between PDIs and NAFLD risk might largely be driven by higher intakes of coffee and tea and lower intakes of sugar-sweetened beverages, fish, and sea foods.

## Discussion

In the present longitudinal study, we found that greater intake of plant-based diets, particularly healthful plant-based diets was associated with lower NAFLD risk and liver fat content, while a higher uPDI was associated with increased NAFLD risk and higher liver fat content. Compared to the lowest quintile, participants in the highest quintile of overall PDI, hPDI, and uPDI had a 22% lower, 26% lower, and 24% higher risk of NAFLD, and 0.51% lower, 0.71% lower, and 0.72% higher liver fat content, respectively.

The longitudinal evidence on associations between plant-based diets and NAFLD risk is scarce, and only several cross-sectional studies reported inconsistent associations between PDIs and NAFLD [12–15]. A study of 18,345 participants in the National Health and Nutrition Examination Survey (NHANES) showed 21% lower, 24% lower, and 34% higher odds of NAFLD for overall PDI, hPDI, and uPDI comparing the highest tertile to the lowest, respectively, in which NAFLD was diagnosed based on the fatty liver index (FLI) [13]. However, in another study of 3900 participants in NHANES, only hPDI showed a significant association with lower transient elastography-diagnosed NAFLD prevalence, and the positive association between uPDI and NAFLD was largely modified and insignificant after further adjusting for BMI [15]. Besides the sample size, the NAFLD diagnosis method might also account for the aforementioned inconsistent findings. Though FLI was frequently used in several large population-based studies to define fatty liver disease (FLD), the more accurate non-invasive measurement of liver steatosis was MRI-PDFF [26]. In another study of 578 participants, the association of three PDIs and the likelihood of MRI-diagnosed FLD was not significant, which might contribute to the limited sample size [14]. In our preregistered analysis, the associations of higher overall PDI and hPDI and lower uPDI with lower NAFLD risk remained significant in fully adjusted model and sensitivity analyses, and the longitudinal study design, the larger sample size, and a more accurate assessment method for intrahepatic liver content increased the confidence in our findings.

The interplay between genetic risk and PDIs has not been reported, and only one study has examined the interaction between the whole diet quality and the overall genetic risk of NAFLD [18]. Based on the Framingham Heart Study, Ma et al. reported that improved diet quality (represented by Mediterranean diet score and AHEI) modified the genetic risk of NAFLD on the liver fat content increase. Though not PDIs, the richness of plant-based foods in the Mediterranean dietary pattern and the good correlation between AHEI and hPDI [30] hinted that PDIs might interplay with NAFLD genetic risk. In our analysis, the significant multiplicative interaction between PDIs and NAFLD-PRS on the risk of NAFLD was observed in hPDI in a sex-specific manner, which might be partly due to the higher NAFLD risk observed in men rather than in women [31].

Several food groups might account for the observed associations, including whole grains, tea, coffee, sugar-sweetened beverages, and red meat. The associations of nuts, tea, and coffee consumption with lower NAFLD risks in our study were in line with previous findings [32, 33]. Those associations might contribute to the higher intake of dietary fibers, flavonoids, caffeine, phytosterols, and plant proteins following a plant-based diet rich in healthy plant-based foods [34], which are all shown to have effects on improved insulin resistance, decreased central obesity, and improved gut microbiome, and hence reduce NAFLD risk [35–39]. The significant mediating effects of BMI on PDIs-NAFLD associations observed in our study also partly supported the aforementioned mechanisms. Besides beneficial foods and nutrients, the positive association between a higher intake of sugar-sweetened beverages and NAFLD risk was in agreement with a previous umbrella review of meta-analysis [40], and the accompanying intake of fructose has proved to promote liver fat accumulation [41]. In addition, previous evidence showed that red meat consumption was associated with increased NAFLD risk [42], but not white meat [43]. In PDIs, red meat and white meat are both grouped into meat, and the inconsistent associations between these two types of meat might explain the marginally significant association of meat with NAFLD risk in our analyses.

The longitudinal study was based on a relatively large sample with a long follow-up period. We used a validated dietary recall method and every dietary assessment information to quantify participants’ dietary intake, which limited the measurement bias. Several limitations should be mentioned. First, the dietary assessment was based on 24-h recall, which might be subjected to recall bias and lead to misclassification. However, the misclassification would likely bias our results toward the null. In addition, the representation of long-term dietary habits was limited, while the results were not substantially changed when we limited our analyses to those who completed at least twice dietary assessments. Second, NAFLD cases were ascertained based on primary care, in-hospital record, and death registry data, which might potentially underestimate the true NAFLD incidence. However, it is unlikely that the underdiagnosed NAFLD cases would be diet-specific. Assuming that the specificity of outcome detection is perfect and sensitivity is lower than 100% in both exposure groups, outcome misclassification would produce little bias in estimating the hazard ratio [44]. Third, though we have controlled the majority of confounders, the potential confounding factors are still likely. Fourth, insulin resistance status in UK Biobank was not available, which interfered with the interpretation of its effect on associations of PDIs with NAFLD risk and liver fat content. However, further adjusting for waist circumference, a marker of central obesity and closely associated with insulin resistance, did not largely change the results. Last, our analyses were conducted among Europeans, limiting our findings’ generalization to other ethnic groups.

## Conclusions

Our results suggested that higher intakes of overall and healthful plant-based diets were associated with lower NAFLD risk regardless of genetic susceptibility. Conversely, an unhealthful plant-based diet was associated with increased NAFLD risk. Our findings highlighted the importance of the quality of plant-based food when adhering to a plant-based dietary pattern to prevent NAFLD in the entire population.

### Supplementary Information

**Additional file 1: Figure S1.** Flow chart of participants included in the present UK Biobank study. **Figure S2.** Priori defined Directed Acyclic Graph. **Figure S3.** Cumulative incidence of NAFLD by quintiles of PDIs. **Figure S4.** Joint associations of three PDIs and genetic risk with NAFLD risk. **Figure S5.** Joint associations of three PDIs and genetic risk with MRI-PDFF. **Figure S6.** Stratified analyses by sex for associations of PDIs quintiles with NAFLD risk and MRI-PDFF. **Table S1.** Example of food items, Filed ID in the UK Biobank, and scoring for plant-based diet indices in 17 food groups. **Table S2.** Definitions of NAFLD in the UK Biobank. **Table S3.** Characteristics of NAFLD-associated SNPs in the UK biobank. **Table S4.** Baseline characteristics between total participants and those with MRI-PDFF data. **Table S5.** Baseline characteristics by NAFLD status. **Table S6.** Associations between plant-based diet indices and MRI-PDFF. **Table S7.** Subgroup analysis of the association between overall PDI and the risk of NAFLD by genetic risk. **Table S8.** Subgroup analysis of the association between hPDI and the risk of NAFLD by genetic risk. **Table S9.** Subgroup analysis of the association between uPDI and the risk of NAFLD by genetic risk. **Table S10.** Hazard ratios (95% confidence intervals) of NAFLD according to sex-specific quintiles of overall plant-based diet index, healthful plant-based diet index, and unhealthful plant-based diet index. **Table S11.** β-coefficient (95% confidence intervals) of MRI-PDFF according to sex-specific quintiles of overall plant-based diet index, healthful plant-based diet index, and unhealthful plant-based diet index. **Table S12.** Subgroup analyses for the associations of PDI, hPDI, and uPDI with the risk of NAFLD per 10-point increment in each index by major confounders. **Table S13.** Subgroup analyses for the associations of PDI, hPDI, and uPDI with MRI-PDFF per 10-point increment in each index by major confounders. **Table S14.** Sensitivity analyses for associations between plant-based diet indices and NAFLD risk. **Table S15.** Mediating effect of BMI on associations between plant-based diet indices and NAFLD risk. **Table S16.** Hazard ratio (95% confidence intervals) for NAFLD according to modified plant-based diet indices (per 10-point increment) with additional adjustment for the excluded food group (servings/day).

## Data Availability

Data from UK Biobank are available to all researchers upon making an application. This research has been conducted using the UK Biobank Resource under Applications 63454 and 69424.

## Abbreviations

AHEI: Alternative Healthy Eating Index
BMI: Body mass index
CI: Confidence interval
FLD: Fatty liver disease
FLI: Fatty liver index
hPDI: Healthful plant-based diet index
HR: Hazards ratio
ICD: International Classification of Diseases
METs: Metabolic equivalent tasks
MRI: Magnetic resonance imaging
NAFLD: Non-alcoholic fatty liver disease
NHANES: National Health and Nutrition Examination Survey
PDFF: Proton density fat fraction
PDI: Plant-based diet index
PRS: Polygenic risk score
SNPs: Single-nucleotide polymorphisms
uPDI: Unhealthful plant-based diet index

## References

[1] Powell EE, Wong VW, Rinella M (2021). Non-alcoholic fatty liver disease. Lancet.

[2] Riazi K, Azhari H, Charette JH, Underwood FE, King JA, Afshar EE (2022). The prevalence and incidence of NAFLD worldwide: a systematic review and meta-analysis. Lancet Gastroenterol Hepatol.

[3] Petermann-Rocha F, Gray SR, Forrest E, Welsh P, Sattar N, Celis-Morales C (2022). Associations of muscle mass and grip strength with severe NAFLD: A prospective study of 333,295 UK Biobank participants. J Hepatol.

[4] Hu FB (2002). Dietary pattern analysis: a new direction in nutritional epidemiology. Curr Opin Lipidol.

[5] Willett W, Rockström J, Loken B, Springmann M, Lang T, Vermeulen S (2019). Food in the Anthropocene: the EAT-Lancet Commission on healthy diets from sustainable food systems. Lancet.

[6] Zelber-Sagi S, Salomone F, Mlynarsky L (2017). The Mediterranean dietary pattern as the diet of choice for non-alcoholic fatty liver disease: Evidence and plausible mechanisms. Liver Int.

[7] Alferink LJM, Erler NS, de Knegt RJ, Janssen HLA, Metselaar HJ, Darwish Murad S (2020). Adherence to a plant-based, high-fibre dietary pattern is related to regression of non-alcoholic fatty liver disease in an elderly population. Eur J Epidemiol.

[8] Yang H, Zhang T, Rayamajhi S, Thapa A, Du W, Meng G (2022). The longitudinal associations between sweet potato intake and the risk of non-alcoholic fatty liver disease: the TCLSIH cohort study. Int J Food Sci Nutr.

[9] Lee D, Chiavaroli L, Ayoub-Charette S, Khan TA, Zurbau A, Au-Yeung F, et al. Important food sources of fructose-containing sugars and non-alcoholic fatty liver disease: a systematic review and meta-analysis of controlled trials. Nutrients. 2022;14(14):2846. 10.3390/nu14142846 PMC9325155 35889803

[10] Park WY, Yiannakou I, Petersen JM, Hoffmann U, Ma J, Long MT (2022). Sugar-sweetened beverage, diet soda, and nonalcoholic fatty liver disease over 6 years: the Framingham Heart Study. Clin Gastroenterol Hepatol.

[11] Satija A, Bhupathiraju SN, Rimm EB, Spiegelman D, Chiuve SE, Borgi L (2016). Plant-based dietary patterns and incidence of type 2 diabetes in US men and women: results from three prospective cohort studies. PLoS Med.

[12] Bhupathiraju SN, Sawicki CM, Goon S, Gujral UP, Hu FB, Kandula NR, et al. A healthy plant-based diet is favorably associated with cardiometabolic risk factors among participants of South Asian ancestry. Am J Clin Nutr. 2022;116(4):1078–90. 10.1093/ajcn/nqac174 PMC9755998 35731596

[13] Mazidi M, Kengne AP (2019). Higher adherence to plant-based diets are associated with lower likelihood of fatty liver. Clin Nutr.

[14] Ratjen I, Morze J, Enderle J, Both M, Borggrefe J, Müller HP (2020). Adherence to a plant-based diet in relation to adipose tissue volumes and liver fat content. Am J Clin Nutr.

[15] Li X, Peng Z, Li M, Zeng X, Li H, Zhu Y, et al. A healthful plant-based diet is associated with lower odds of nonalcoholic fatty liver disease. Nutrients. 2022;14(19):4099. 10.3390/nu14194099 PMC9572274 36235752

[16] Anstee QM, Seth D, Day CP (2016). Genetic factors that affect risk of alcoholic and nonalcoholic fatty liver disease. Gastroenterology.

[17] Trépo E, Valenti L (2020). Update on NAFLD genetics: from new variants to the clinic. J Hepatol.

[18] Ma J, Hennein R, Liu C, Long MT, Hoffmann U, Jacques PF (2018). Improved diet quality associates with reduction in liver fat, particularly in individuals with high genetic risk scores for nonalcoholic fatty liver disease. Gastroenterology.

[19] Liu B, Young H, Crowe FL, Benson VS, Spencer EA, Key TJ (2011). Development and evaluation of the Oxford WebQ, a low-cost, web-based method for assessment of previous 24 h dietary intakes in large-scale prospective studies. Public Health Nutr.

[20] Greenwood DC, Hardie LJ, Frost GS, Alwan NA, Bradbury KE, Carter M (2019). Validation of the Oxford WebQ Online 24-Hour Dietary Questionnaire Using Biomarkers. Am J Epidemiol.

[21] Satija A, Bhupathiraju SN, Spiegelman D, Chiuve SE, Manson JE, Willett W (2017). Healthful and unhealthful plant-based diets and the risk of coronary heart disease in U.S. adults. J Am Coll Cardiol..

[22] Population-level risks of alcohol consumption by amount (2022). geography, age, sex, and year: a systematic analysis for the Global Burden of Disease Study 2020. Lancet.

[23] Hagström H, Adams LA, Allen AM, Byrne CD, Chang Y, Grønbaek H (2021). Administrative coding in electronic health care record-based research of NAFLD: an expert panel consensus statement. Hepatology.

[24] Wilman HR, Kelly M, Garratt S, Matthews PM, Milanesi M, Herlihy A (2017). Characterisation of liver fat in the UK Biobank cohort. PLoS One.

[25] Caussy C, Alquiraish MH, Nguyen P, Hernandez C, Cepin S, Fortney LE (2018). Optimal threshold of controlled attenuation parameter with MRI-PDFF as the gold standard for the detection of hepatic steatosis. Hepatology.

[26] Tamaki N, Ajmera V, Loomba R (2022). Non-invasive methods for imaging hepatic steatosis and their clinical importance in NAFLD. Nat Rev Endocrinol.

[27] Parisinos CA, Wilman HR, Thomas EL, Kelly M, Nicholls RC, McGonigle J (2020). Genome-wide and Mendelian randomisation studies of liver MRI yield insights into the pathogenesis of steatohepatitis. J Hepatol.

[28] Bycroft C, Freeman C, Petkova D, Band G, Elliott LT, Sharp K (2018). The UK Biobank resource with deep phenotyping and genomic data. Nature.

[29] Blane D, Townsend P, Phillimore P, Beattie A (1987). Health and deprivation: inequality and the North. Br J Sociol.

[30] Shan Z, Li Y, Baden MY, Bhupathiraju SN, Wang DD, Sun Q (2020). Association between healthy eating patterns and risk of cardiovascular disease. JAMA Intern Med.

[31] Lonardo A, Nascimbeni F, Ballestri S, Fairweather D, Win S, Than TA (2019). Sex differences in nonalcoholic fatty liver disease: state of the art and identification of research gaps. Hepatology.

[32] Zhang S, Fu J, Zhang Q, Liu L, Meng G, Yao Z (2019). Association between nut consumption and non-alcoholic fatty liver disease in adults. Liver Int.

[33] Chhimwal J, Patial V, Padwad Y (2021). Beverages and Non-alcoholic fatty liver disease (NAFLD): think before you drink. Clin Nutr.

[34] Mozaffarian D (2016). Dietary and policy priorities for cardiovascular disease, diabetes, and obesity: a comprehensive review. Circulation.

[35] Zhao H, Yang A, Mao L, Quan Y, Cui J, Sun Y (2020). Association between dietary fiber intake and non-alcoholic fatty liver disease in adults. Front Nutr.

[36] Van De Wier B, Koek GH, Bast A, Haenen GR (2017). The potential of flavonoids in the treatment of non-alcoholic fatty liver disease. Crit Rev Food Sci Nutr.

[37] Xin X, Cheng C, Bei-Yu C, Hong-Shan L, Hua-Jie T, Xin W (2021). Caffeine and EGCG alleviate high-trans fatty acid and high-carbohydrate diet-induced NASH in mice: commonality and specificity. Front Nutr.

[38] Yang JW, Ji HF. Phytosterols as bioactive food components against nonalcoholic fatty liver disease. Crit Rev Food Sci Nutr. 2023;63(20):4675–86. 10.1080/10408398.2021.2006137 34871105

[39] Markova M, Pivovarova O, Hornemann S, Sucher S, Frahnow T, Wegner K (2017). Isocaloric diets high in animal or plant protein reduce liver fat and inflammation in individuals with type 2 diabetes. Gastroenterology.

[40] Xia Y, Wu Q, Dai H, Lv J, Liu Y, Sun H (2021). Associations of nutritional, lifestyle, and metabolic factors with non-alcoholic fatty liver disease: an umbrella review with more than 380,000 participants. Front Nutr.

[41] Jensen T, Abdelmalek MF, Sullivan S, Nadeau KJ, Green M, Roncal C (2018). Fructose and sugar: a major mediator of non-alcoholic fatty liver disease. J Hepatol.

[42] Noureddin M, Zelber-Sagi S, Wilkens LR, Porcel J, Boushey CJ, Le Marchand L (2020). Diet associations with nonalcoholic fatty liver disease in an ethnically diverse population: the multiethnic cohort. Hepatology.

[43] Hashemian M, Merat S, Poustchi H, Jafari E, Radmard AR, Kamangar F (2021). Red meat consumption and risk of nonalcoholic fatty liver disease in a population with low meat consumption: the Golestan Cohort Study. Am J Gastroenterol.

[44] Rothman KJ GS, Lash TL. Modern epidemiology. 3rd ed. Philadelphia: Wolters Kluwer Health/Lippincott Williams & Wilkins; 2008.

