# Supplementary material for: Plant-based diets, genetic predisposition and risk of non-alcoholic fatty liver disease
Source: BMC Med. 2023 Sep 12;21:351. doi: 10.1186/s12916-023-03028-w (PMC10496397; doi:10.1186/s12916-023-03028-w)
Supplement: Supplementary file 1 — Additional file 1: Figure S1. Flow chart of participants included in the present UK Biobank study. Figure S2. Priori defined Directed Acyclic Graph. Figure S3. Cumulative incidence of NAFLD by quintiles of PDIs. Figure S4. Joint associations of three PDIs and genetic risk with NAFLD risk. Figure S5. Joint associations of three PDIs and genetic risk with MRI-PDFF. Figure S6. Stratified analyses by sex for associations of PDIs quintiles with NAFLD risk and MRI-PDFF. Table S1. Example of food items, Filed ID in the UK Biobank, and scoring for plant-based diet indices in 17 food groups. Table S2. Definitions of NAFLD in the UK Biobank. Table S3. Characteristics of NAFLD-associated SNPs in the UK biobank. Table S4. Baseline characteristics between total participants and those with MRI-PDFF data. Table S5. Baseline characteristics by NAFLD status. Table S6. Associations between plant-based diet indices and MRI-PDFF. Table S7. Subgroup analysis of the association between overall PDI and the risk of NAFLD by genetic risk. Table S8. Subgroup analysis of the association between hPDI and the risk of NAFLD by genetic risk. Table S9. Subgroup analysis of the association between uPDI and the risk of NAFLD by genetic risk. Table S10. Hazard ratios (95% confidence intervals) of NAFLD according to sex-specific quintiles of overall plant-based diet index, healthful plant-based diet index, and unhealthful plant-based diet index. Table S11. β-coefficient (95% confidence intervals) of MRI-PDFF according to sex-specific quintiles of overall plant-based diet index, healthful plant-based diet index, and unhealthful plant-based diet index. Table S12. Subgroup analyses for the associations of PDI, hPDI, and uPDI with the risk of NAFLD per 10-point increment in each index by major confounders. Table S13. Subgroup analyses for the associations of PDI, hPDI, and uPDI with MRI-PDFF per 10-point increment in each index by major confounders. Table S14. Sensitivity analyses for associations between [file 12916_2023_3028_MOESM1_ESM.docx]

**Additional file 1**

**Figure S1.** Flow chart of participants included in the present UK Biobank study

**Figure S2.** Priori defined Directed Acyclic Graph

**Figure S3.** Cumulative incidence of NAFLD by quintiles of PDIs.

**Figure S4.** Joint associations of three PDIs and genetic risk with NAFLD risk

**Figure S5.** Joint associations of three PDIs and genetic risk with MRI-PDFF

**Figure S6.** Stratified analyses by sex for associations of PDIs quintiles with NAFLD risk and MRI-PDFF

**Table S1.** Example of food items, Filed ID in the UK Biobank, and scoring for plant-based diet indices in 17 food groups

**Table S2.** Definitions of NAFLD in the UK Biobank

**Table S3.** Characteristics of NAFLD-associated SNPs in the UK biobank

**Table S4.** Baseline characteristics between total participants and those with MRI-PDFF data

**Table S5.** Baseline characteristics by NAFLD status

**Table S6.** Associations between plant-based diet indices and MRI-PDFF

**Table S7.** Subgroup analysis of the association between overall PDI and the risk of NAFLD by genetic risk

**Table S8.** Subgroup analysis of the association between hPDI and the risk of NAFLD by genetic risk

**Table S9.** Subgroup analysis of the association between uPDI and the risk of NAFLD by genetic risk

**Table S10.** Hazard ratios (95% confidence intervals) of NAFLD according to sex-specific quintiles of overall plant-based diet index, healthful plant-based diet index, and unhealthful plant-based diet index

**Table S11.** β-coefficient (95% confidence intervals) of MRI-PDFF according to sex-specific quintiles of overall plant-based diet index, healthful plant-based diet index, and unhealthful plant-based diet index

**Table S12.** Subgroup analyses for the associations of PDI, hPDI, and uPDI with the risk of NAFLD per 10-point increment in each index by major confounders

**Table S13.** Subgroup analyses for the associations of PDI, hPDI, and uPDI with MRI-PDFF per 10-point increment in each index by major confounders

**Table S14.** Sensitivity analyses for associations between plant-based diet indices and NAFLD risk

**Table S15.** Mediating effect of BMI on associations between plant-based diet indices and NAFLD risk

**Table S16.** Hazard ratio (95% confidence intervals) for NAFLD according to modified plant-based diet indices (per 10-point increment) with additional adjustment for the excluded food group (servings/day)


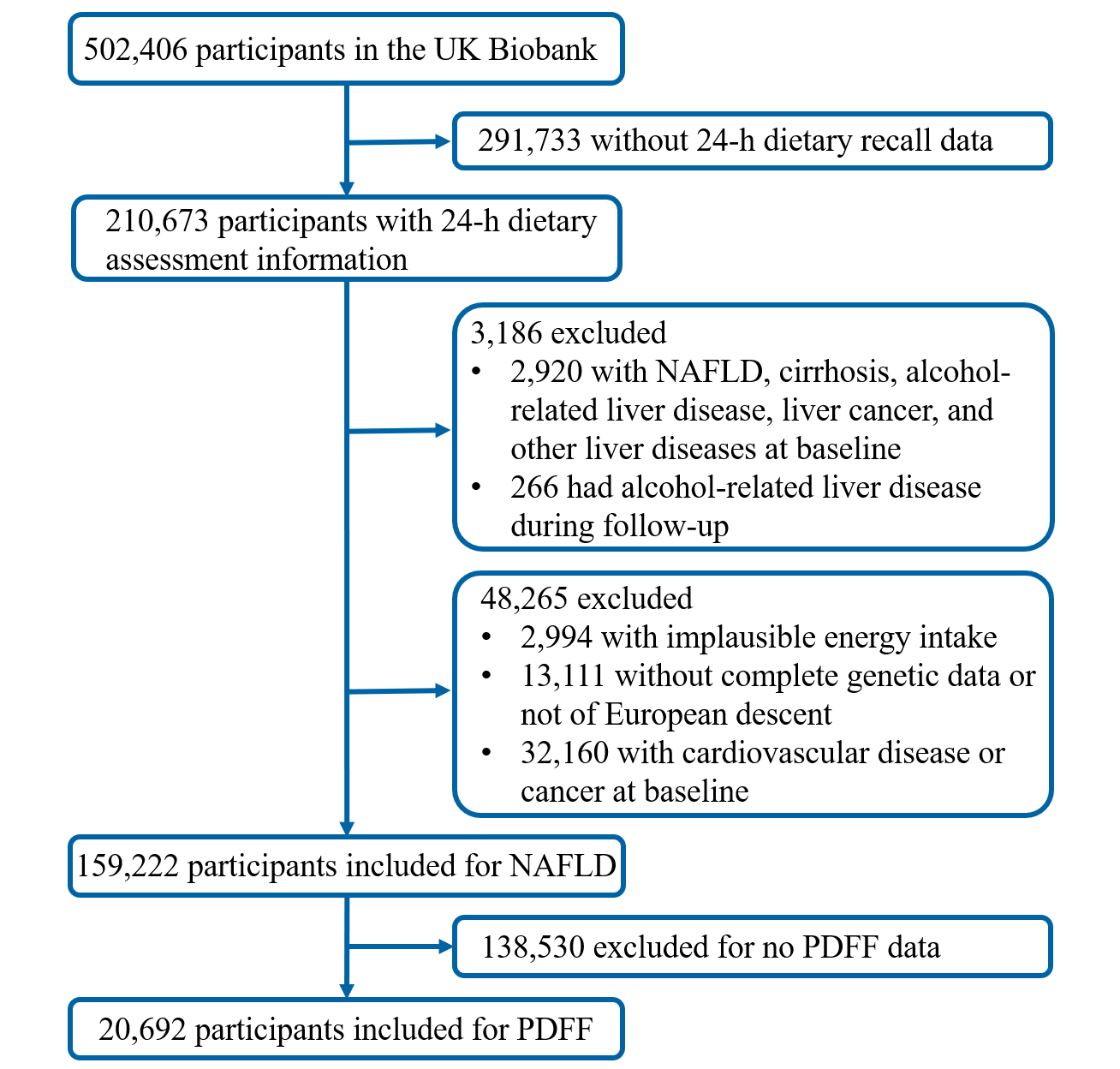


**Figure S1. Flow chart of participants included in the present UK Biobank study**

Abbreviations: NAFLD, non-alcoholic fatty liver disease; PDFF, proton density fat fraction;

**
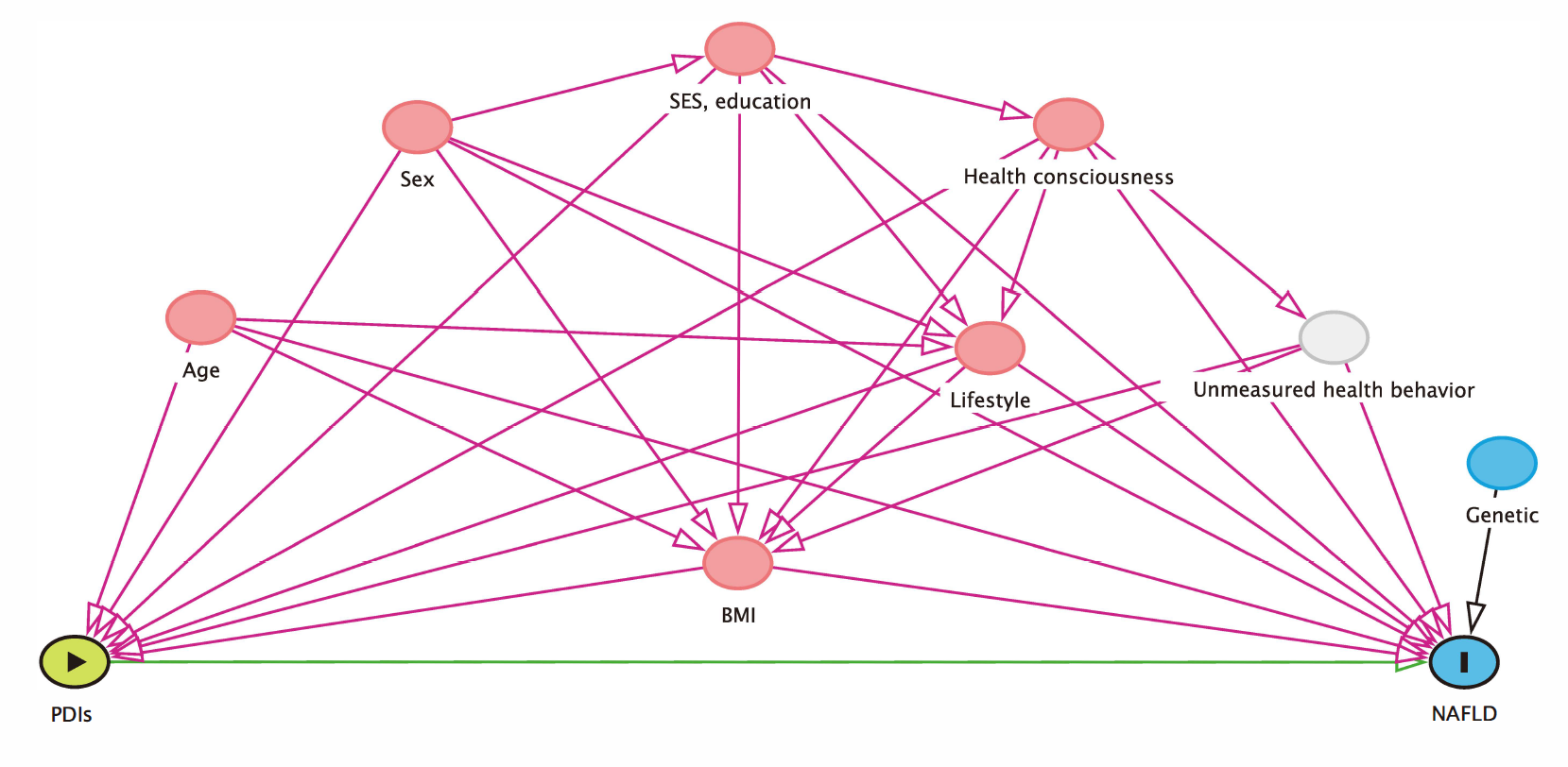
**

**Figure S2. Priori defined Directed Acyclic Graph**

The directed acyclic graph above was generated via an open-source application DAGitty based on R package 'dagitty' ( <http://dagitty.net/dags.html>).

Reference: Johannes Textor, Benito van der Zander, Mark K. Gilthorpe, Maciej Liskiewicz, George T.H. Ellison. Robust causal inference using directed acyclic graphs: the R package 'dagitty'. International Journal of Epidemiology 45(6):1887-1894, 2016. https://doi.org/10.1093/ije/dyw341.

Abbreviations: BMI, body mass index; NAFLD, non-alcoholic fatty liver disease; PDIs, plant-based diet indices; SES; socioeconomic status

**
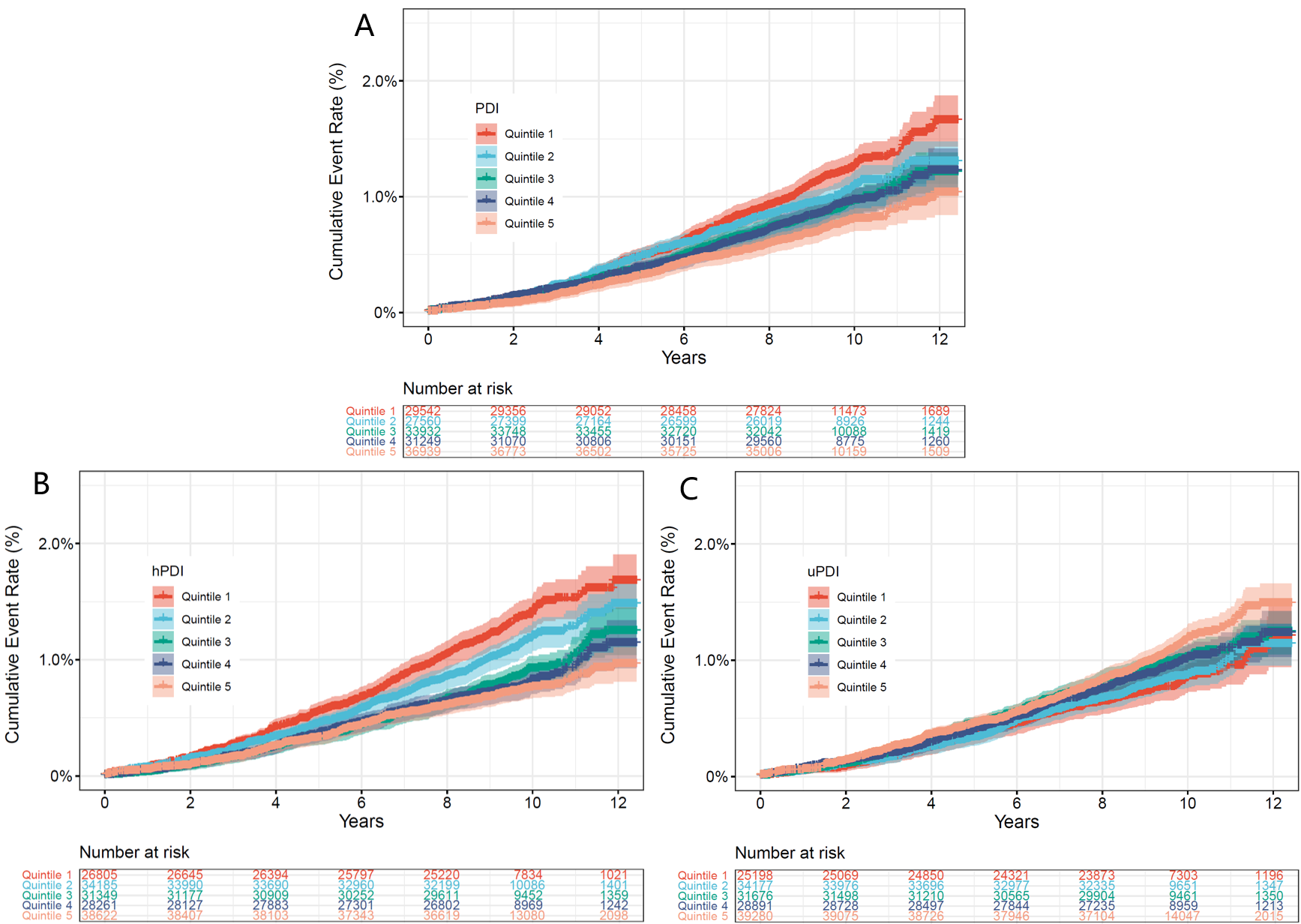
**

**Figure S3. Cumulative incidence of NAFLD by quintiles of PDIs.**

A: Cumulative incidence of NAFLD by quintiles of overall PDI

B: Cumulative incidence of NAFLD by quintiles of hPDI

C: Cumulative incidence of NAFLD by quintiles of uPDI

Abbreviations: hPDI, healthful plant-based diet index; NAFLD, non-alcoholic fatty liver disease; PDI, plant-based diet index; uPDI, unhealthful plant-based diet index.

**
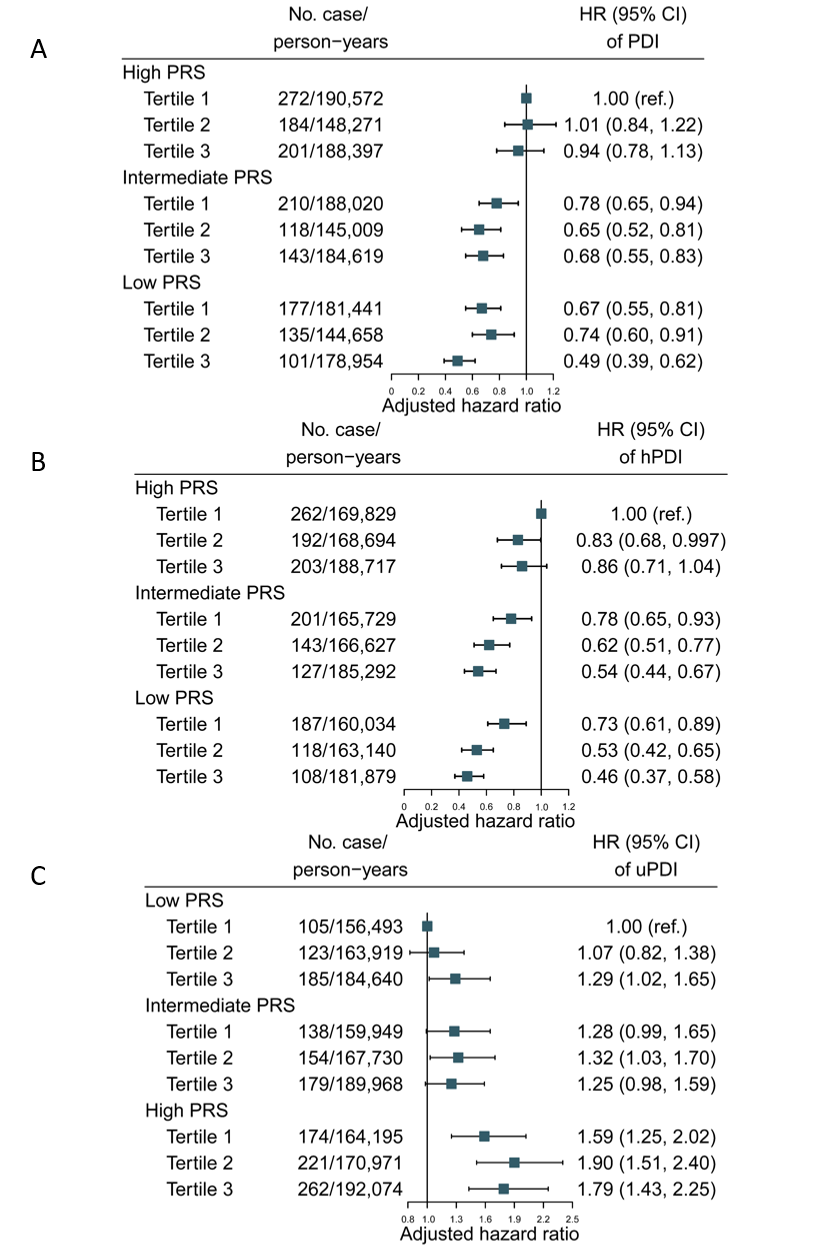
**

**Figure S4. Joint associations of plant-based diet indices and PRS with NAFLD risk**

Adjusted for age at the last dietary assessment, sex, education, household income, Townsend deprivation index, assessment centers, smoking, alcohol consumption, physical activity, total energy, BMI, first 10 principal components of ancestry, and genotype measurement batch.

A: Joint association of overall PDI and PRS with NAFLD risk.

B: Joint association of hPDI and PRS with NAFLD risk

C: Joint association of uPDI and PRS with NAFLD risk

Abbreviations: BMI, body mass index; CI, confidence interval; hPDI, healthful plant-based diet index; HR, hazards ratio; NAFLD, non-alcoholic fatty liver disease; PDI, plant-based diet index; PRS, polygenic risk score; ref., reference; uPDI, unhealthful plant-based diet index.

**
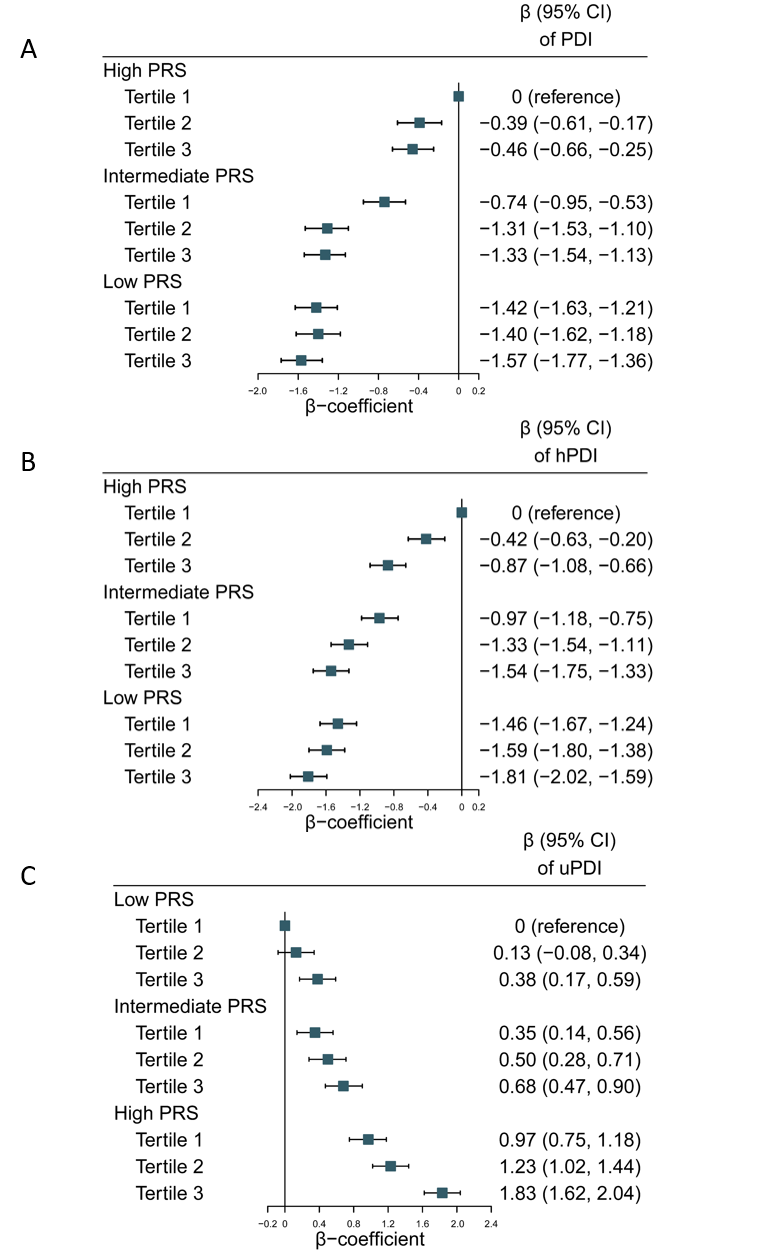
**

**Figure S5. Joint associations of plant-based diet indices and PRS with MRI-PDFF**

Adjusted for age at the last dietary assessment, age at MRI scan, sex, education, household income, Townsend deprivation index, assessment centers, smoking, alcohol consumption, physical activity, total energy, BMI, first 10 principal components of ancestry, and genotype measurement batch.

A: Joint association of overall PDI and PRS with MRI-PDFF.

B: Joint association of hPDI and PRS with MRI-PDFF

C: Joint association of uPDI and PRS with MRI-PDFF

Abbreviations: BMI, body mass index; CI, confidence interval; hPDI, healthful plant-based diet index; MRI, magnetic resonance imaging; NAFLD, non-alcoholic fatty liver disease; PDFF, proton density fat fraction; PDI, plant-based diet index; PRS, polygenic risk score; uPDI, unhealthful plant-based diet index.

**
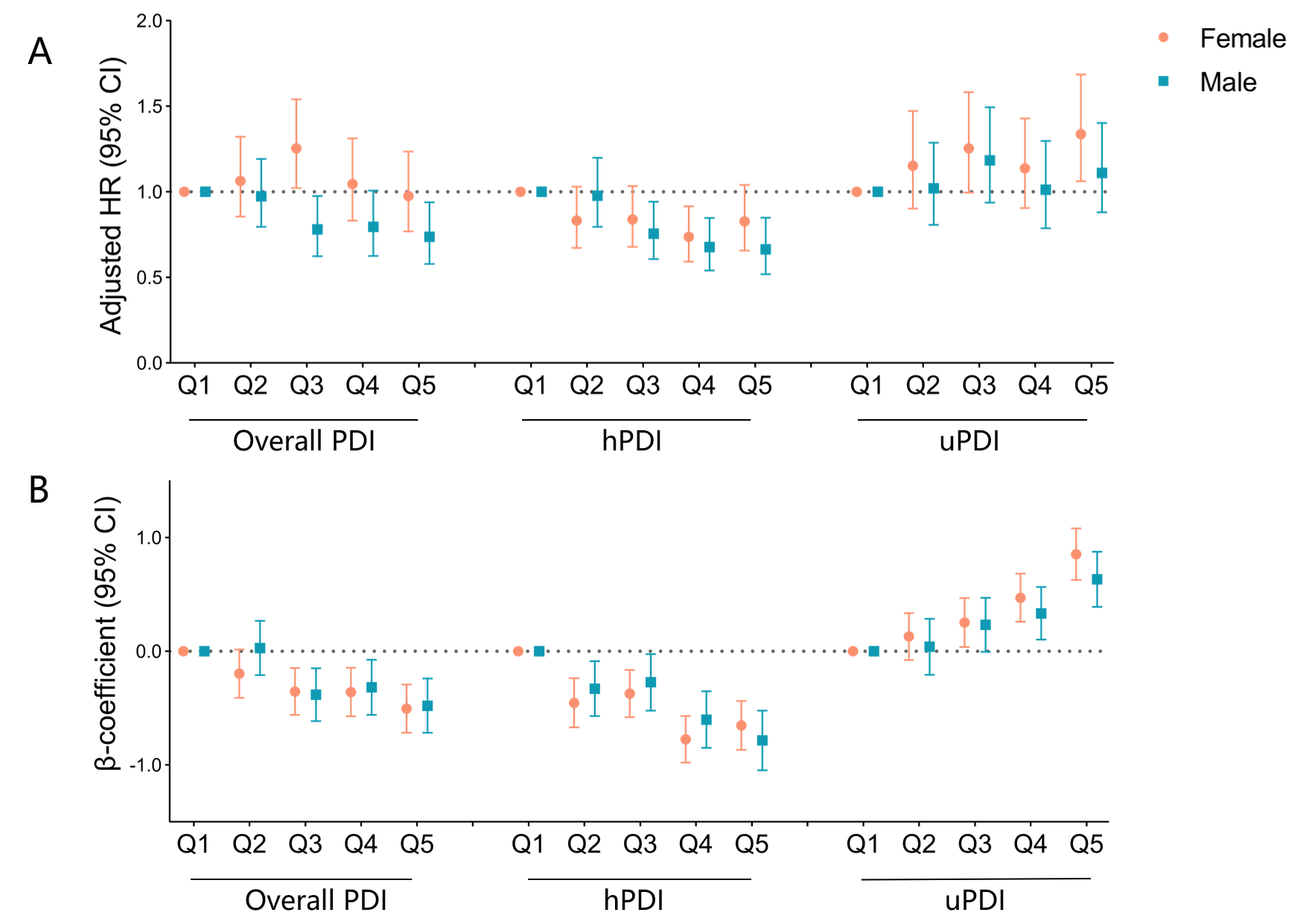
**

**Figure S6. Stratified analyses by sex for associations of PDIs quintiles with NAFLD risk and MRI-PDFF**

Adjusted for age at the last dietary assessment, age at MRI scan (for MRI-PDFF only), sex, education, household income, Townsend deprivation index, assessment centers, smoking, alcohol consumption, physical activity, total energy, BMI, PRS, first 10 principal components of ancestry, and genotype measurement batch.

A: Hazard ratios (95% CI) for NAFLD risk according to PDIs quintiles by sex

B: β-coefficient (95% CI) for MRI-PDFF according to PDIs quintiles by sex

Abbreviations: BMI, body mass index; CI, confidence interval; hPDI, healthful plant-based diet index; MRI, magnetic resonance imaging; NAFLD, non-alcoholic fatty liver disease; PDFF, proton density fat fraction; PDI, plant-based diet index; PRS, polygenic risk score; uPDI, unhealthful plant-based diet index.

**Table S1. Example of food items, Filed ID in the UK Biobank, and scoring for** **plant-based diet indices in 17 food groups**

| **Food groups** | **Food items** | **Field ID** | **Scoring for PDI** | **Scoring for hPDI** | **Scoring for uPDI** |
| --- | --- | --- | --- | --- | --- |
| **Plant-based food groups** | | | | | |
| **Healthy** |  |  |  |  |  |
| Whole grains | Porridge, muesli, plain cereal, bran cereal, whole-wheat cereal, oatcakes, wholemeal bread (flour type: wholemeal), whole meal pasta, brown rice, couscous, other cooked grains (such as bulgur). | 100770, 100800, 100830, 100840, 100850, 100950, 20091, 101020, 20092, 101090, 20093, 101160, 20094, 101260, 102720, 102740, 102780 | Positive | Positive | Reverse |
| Fruits | Stewed fruit, prune, dried fruit, mixed fruit, apple, banana, berries, cherries, grapefruit, grapes, mango, melon, orange, orange-like small fruits, peach/nectarine, pear, pineapple, plum, other fruits, olives | 104410, 104420, 104430, 104440, 104450, 104460, 104470, 104480, 104490, 104500, 104510, 104520, 104530, 104540, 104550, 104560, 104570, 104580, 104590, 102490 | Positive | Positive | Reverse |
| Vegetables | Mixed vegetables, vegetable pieces, coleslaw, side salad, avocado, beetroot, broccoli, butternut squash, cabbage/kale, carrots, cauliflower, celery, courgette, cucumber, garlic, leeks, lettuce, mushrooms, onion, parsnip, sweet peppers, spinach, sprouts, sweetcorn, fresh tomatoes, cooked or tinned tomatoes, turnip/swede, watercress, other vegetable intake | 104060, 104070, 104080, 104090, 104100, 104130, 104140, 104150, 104160, 104170, 104180, 104190, 104200, 104210, 104220, 104230, 104240, 104250, 104260, 104270, 104290, 104300, 104310, 104320, 104340, 104350, 104360, 104370, 104380 | Positive | Positive | Reverse |
| Nuts | Salted peanuts, unsalted peanuts, salted nuts, unsalted nuts, seeds | 102410, 102420, 102430, 102440, 102450 | Positive | Positive | Reverse |
| Legumes | Vegetarian sausages/burgers, tofu, quorn, other vegetarian alternative, baked beans, pulse, broad beans, green beans, peas | 103260, 103270, 103280, 103290, 104000, 104010, 104110, 104120, 104280 | Positive | Positive | Reverse |
| Tea and coffee | Instant coffee, filtered coffee, cappuccino, latte, espresso, other coffee drinks, standard tea, rooibos tea, green tea, herbal tea, other tea, low calorie hot chocolate, hot chocolate | 100250, 100270, 100290, 100300, 100310, 100330, 100400, 100410, 100420, 100430, 100440, 100540, 100550 | Positive | Positive | Reverse |
| **Less healthy** |  |  |  |  |  |
| Refined grains | Oat crunch, sweetened cereal, other cereal, white bread (flour types: white, mix, and others), naan bread, garlic bread, crispbread, other bread, white pasta, white rice, snackpot, couscous, pancake, scotch pancake, croissant, scone, savoury biscuits, cheesy biscuits, sweet potato, other savoury snack | 100810, 100820, 100860, 101230, 101240, 101250, 101270, 102710, 102730, 102760, 102770, 102010, 102020, 102050, 102070, 102470, 102480, 102500, 104330 | Positive | Reverse | Positive |
| Potatoes | Fried potatoes, boiled/baked potatoes, mashed potatoes, crisps (e.g., potato chips) | 102460, 104020, 104030, 104050 | Positive | Reverse | Positive |
| Sugary drinks | Low calorie or diet drinks (e.g. fizzy, squash), carbonated (fizzy) drinks, squash or cordial | 100160, 100170, 100180 | Positive | Reverse | Positive |
| Fruit juices | Orange juice, grapefruit juice, other fruit/vegetable juice, fruit smoothie | 100190, 100200, 100210, 100220 | Positive | Reverse | Positive |
| Sweets and desserts | Double crust pie, single crust pie/flan, crumble topping, Yorkshire pudding, Danish pastry, fruitcake, cake, doughnuts, sponge pudding, other dessert, chocolate bar, white chocolate, milk chocolate, dark chocolate, chocolate-covered raisin, chocolate sweet, diet sweets, chocolate-covered biscuits, chocolate biscuits, sweet biscuits, other sweets | 101970, 101980, 101990, 102030, 102060, 102170, 102180, 102190, 102200, 102210, 102230, 102260, 102270, 102280, 102290, 102300, 102310, 102320, 102330, 102340, 102350, 102360, 102370, 102380 | Positive | Reverse | Positive |
| **Animal-based food groups** | | | | | |
| Animal fat | Butter on bread/crackers (spreadable, low fat, normal fat, or unknown type), dairy spread on bread/crackers (very low fat, low fat, normal fat, unknown type) | 101310, 101350, 101390, 101430, 101470, 101510, 101550 | Reverse | Reverse | Reverse |
| Dairy | Milk, dairy smoothie, flavored milk, yogurt, ice-cream, cheesecake, milk-based pudding, other milk-based pudding, low fat hard cheese, hard cheese, soft cheese, blue cheese, low fat cheese spread, cheese spread, cottage cheese, feta cheese, mozzarella cheese, goat's cheese, other cheese | 100230, 100520, 100530, 102090, 102120, 102140, 102150, 102220, 102810, 102820, 102830, 102840, 102850, 102860, 102870, 102880, 102890, 102900, 102910 | Reverse | Reverse | Reverse |
| Eggs | Whole eggs, omelettes or scrambled egg, eggs in sandwiches, scotch egg, other egg dishes | 102940, 102950, 102960, 102970, 102980 | Reverse | Reverse | Reverse |
| Fish or seafood | Tinned tuna, oily fish, breaded fish, battered fish, white fish, prawns, lobster/crab, shellfish, other fish intake | 103150, 103160, 103170, 103180, 103190, 103200, 103210, 103220, 103230 | Reverse | Reverse | Reverse |
| Meat | Sausage, beef, pork, lamb, crumbed or deep-fried poultry, poultry, bacon, ham, liver, other meat intake | 103010, 103020, 103030, 103040, 103050, 103060, 103070, 103080, 103090, 103100 | Reverse | Reverse | Reverse |
| Miscellaneous animal-based foods | Pizza, Indian snacks | 102000, 102040 | Reverse | Reverse | Reverse |

Abbreviations: hPDI, healthful plant-based diet index; PDI, plant-based diet index; uPDI, unhealthful plant-based diet index.

**Table S2. Definitions of NAFLD in the UK Biobank**

|  | **ICD-9** | **ICD-10** | **Primary care** |
| --- | --- | --- | --- |
| **NAFLD** | 5718 | K760 | J61y., J61y0, J61y1, J61y2, J61y7, J61y9, J61yz (Read V2); J61y., J61y0, J61y1, J61y2, J61y7, J61yz, X307v, X307W, XE2Zz (Read CTV3) |

Abbreviations: NAFLD, non-alcoholic fatty liver disease; ICD, International Classification of Diseases.

**Table S3. Characteristics of NAFLD-associated SNPs in the UK biobank**

| **SNP** | **Chr** | **Gene** | **Risk allele** | **Reference allele** | **Beta** |
| --- | --- | --- | --- | --- | --- |
| rs738409 | 22 | PNPLA3 | G | C | 0.594 |
| rs58542926 | 19 | TM6SF2 | T | C | 0.166 |
| rs641738 | 19 | MBOAT7 | T | C | 0.073 |
| rs1260326 | 2 | GCKR | T | C | 0.271 |
| rs72613567 | 4 | HSD17B13 | T | TA | 0.216 |

Abbreviations: Chr, chromosome; NAFLD, non-alcoholic fatty liver disease; SNP, single nucleotide polymorphisms.

**Table S4. Baseline characteristics between total participants and those with MRI-PDFF data**

| Characteristics | Total participants (n=159,222) | With MRI-PDFF data (n=20,692) |
| --- | --- | --- |
| Age, mean (SD), y | 58.0 (8.0) | 57.5 (7.4) |
| Male, n (%) | 10,545 (44.3) | 9,815 (47.4) |
| Townsend Deprivation Index, median (IQR) | -2.4 (-3.8, -0.1) | -2.7 (-3.9, -0.6) |
| Deprivation fifth, n (%) |  |  |
| First (least deprived) | 31,807 (20.0) | 4,691 (22.7) |
| Second to fourth | 95,416 (59.9) | 12,524 (60.5) |
| Fifth (most deprived) | 31,804 (20.0) | 3,462 (16.7) |
| Unknown | 195 (0.1) | 15 (0.1) |
| Education |  |  |
| College or university | 68,790 (43.2) | 10,422 (50.4) |
| Vocational | 15,997 (10.1) | 1,913 (9.3) |
| Upper secondary | 21,397 (13.4) | 2,861 (13.8) |
| Lower secondary | 40,041 (25.2) | 4,520 (21.8) |
| Others | 12,419 (7.8) | 942 (4.6) |
| Unknown | 578 (0.4) | 34 (0.2) |
| Household income, £ |  |  |
| <18,000 | 20,202 (12.7) | 1,935 (9.4) |
| 18,000-30,999 | 33,545 (21.1) | 4,027 (19.5) |
| 31,000-51,999 | 41,687 (26.2) | 5,849 (28.3) |
| 52,000-100,000 | 37,108 (23.3) | 5,753 (27.8) |
| >100,000 | 11,114 (7.0) | 1,587 (7.7) |
| Unknown | 15,566 (9.8) | 1,541 (7.5) |
| Alcohol consumption among drinkers, median (IQR), g/day | 11.9 (5.1, 21.6) | 12.4 (6.0, 22.7) |
| Current smoker, n (%) | 12,144 (7.6) | 1,154 (5.6) |
| Body mass index, mean (SD), kg/m^2^ | 26.8 (4.6) | 26.3 (4.1) |
| Waist circumference, mean (SD), cm | 88.5 (13.1) | 87.4 (12.5) |
| Total physical activity, MET-mins/week |  |  |
| 0-599 | 24,064 (15.1) | 3,268 (15.8) |
| 600-1199 | 24,933 (15.7) | 3,373 (16.3) |
| ≥1200 | 86,396 (54.3) | 11,416 (55.2) |
| Unknown | 23,829 (15.0) | 2,635 (12.7) |
| Energy intake (kcal/d) | 2,002.1 (1,679.1, 2,370.0) | 2,028.5 (1,721.0, 2,379.3) |
| Alanine aminotransferase, median (IQR), U/L | 19.6 (15.1, 26.5) | 19.5 (15.0, 26.3) |
| Albumin, median (IQR), g/L | 45.4 (43.7, 47.1) | 45.4 (43.8, 47.1) |
| Gamma glutamyltransferase, median (IQR), U/L | 24.5 (17.6, 37.6) | 23.7 (17.2, 36.1) |
| Glucose, mean (SD), mmol/L | 5.1 (1.1) | 5.0 (1.0) |
| Glycated haemoglobin, mean (SD), mmol/mol | 35.3 (5.6) | 34.8 (5.0) |
| Triglycerides, median (IQR), mmol/L | 125.5 (89.2, 181.3) | 121.5 (86.6, 175.7) |
| Depression, n (%) | 21,710 (13.6) | 2,422 (11.7) |
| Dyslipidemia, n (%) | 74,740 (46.9) | 9,078 (43.9) |
| Hypertension, n (%) | 79,218 (49.8) | 9,340 (45.4) |
| Diabetes, n (%) | 6,392 (4.0) | 638 (3.1) |
| PDI, median (IQR) | 50 (47, 54) | 51 (47, 54) |
| hPDI, median (IQR) | 54 (50, 58) | 54 (50, 58) |
| uPDI, median (IQR) | 55 (51, 59) | 55 (51, 59) |

Abbreviations: hPDI, healthful plant-based diet index; IQR, interquartile range; MRI, magnetic resonance imaging; PDFF, proton density fat fraction; PDI, plant-based diet index; SD, standard deviation; uPDI, unhealthful plant-based diet index.

**Table S5. Baseline characteristics by NAFLD status**

| Characteristics | Non NAFLD cases (n=157,681) | NAFLD cases (n=1,541) |
| --- | --- | --- |
| Age, mean (SD), y | 58.0 (8.0) | 58.0 (7.9) |
| Male, n (%) | 69,792 (44.3) | 753 (48.9) |
| Townsend Deprivation Index, median (IQR) | -2.4 (-3.8, -0.1) | -2.7 (-3.9, -0.6) |
| Deprivation fifth, n (%) |  |  |
| First (least deprived) | 31,561 (20.0) | 246 (16.0) |
| Second to fourth | 94,549 (60.0) | 867 (56.3) |
| Fifth (most deprived) | 31,379 (19.9) | 425 (27.6) |
| Unknown | 192 (0.1) | 3 (0.2) |
| Education |  |  |
| College or university | 68,284 (43.3) | 506 (32.8) |
| Vocational | 15,793 (10.0) | 204 (13.2) |
| Upper secondary | 21,195 (13.4) | 202 (13.1) |
| Lower secondary | 39,608 (25.1) | 433 (28.1) |
| Others | 12,230 (7.8) | 189 (12.3) |
| Unknown | 571 (0.4) | 7 (0.5) |
| Household income, £ |  |  |
| <18,000 | 19,946 (12.7) | 256 (16.6) |
| 18,000-30,999 | 33,162 (21.0) | 383 (24.9) |
| 31,000-51,999 | 41,299 (26.2) | 388 (25.2) |
| 52,000-100,000 | 36,826 (23.4) | 282 (18.3) |
| >100,000 | 11,036 (7.0) | 78 (5.1) |
| Unknown | 15,412 (9.8) | 154 (10.0) |
| Alcohol consumption among drinkers, median (IQR), g/day | 11.9 (5.1, 21.5) | 10.6 (3.4, 25.5) |
| Current smoker, n (%) | 11971 (7.6) | 173 (11.2) |
| Body mass index, mean (SD), kg/m^2^ | 26.7 (4.5) | 30.9 (5.4) |
| Waist circumference, mean (SD), cm | 88.4 (13.1) | 99.5 (13.2) |
| Total physical activity, MET-mins/week |  |  |
| 0-599 | 23,701 (15.0) | 363 (23.6) |
| 600-1199 | 24,713 (15.7) | 220 (14.3) |
| ≥1200 | 85,709 (54.4) | 687 (44.6) |
| Unknown | 23,558 (14.9) | 271 (17.6) |
| Energy intake (kcal/d) | 2,001.9 (1,679.2, 2,369.5) | 2,018.0 (1,652.8, 2,420.3) |
| Alanine aminotransferase, median (IQR), U/L | 19.5 (15.0, 26.4) | 29.6 (20.7, 43.8) |
| Albumin, median (IQR), g/L | 45.4 (43.7, 47.1) | 45.5 (43.6, 47.3) |
| Gamma glutamyltransferase, median (IQR), U/L | 24.4 (17.5, 37.4) | 41.8 (26.8, 71.7) |
| Glucose, mean (SD), mmol/L | 5.1 (1.0) | 5.5 (1.8) |
| Glycated haemoglobin, mean (SD), mmol/mol | 35.2 (5.6) | 37.9 (8.8) |
| Triglycerides, median (IQR), mmol/L | 125.1 (89.0, 180.7) | 172.1 (122.0, 243.0) |
| Depression, n (%) | 21366 (13.6) | 344 (22.3) |
| Dyslipidemia, n (%) | 73,596 (46.7) | 1,144 (74.2) |
| Hypertension, n (%) | 78,175 (49.6) | 1,043 (67.7) |
| Diabetes, n (%) | 6,165 (3.9) | 227 (14.7) |
| PDI, median (IQR) | 51 (47, 54) | 50 (46, 53) |
| hPDI, median (IQR) | 54 (50, 58) | 53 (49, 57) |
| uPDI, median (IQR) | 55 (51, 59) | 56 (52, 60) |

Abbreviations: hPDI, healthful plant-based diet index; IQR, interquartile range; MRI, magnetic resonance imaging; PDFF, proton density fat fraction; PDI, plant-based diet index; SD, standard deviation; uPDI, unhealthful plant-based diet index.

**Table S6. Associations between plant-based diet indices and MRI-PDFF**

|  | Quintile of dietary score | | | | | *P* for trend | per 10-point increment |
| --- | --- | --- | --- | --- | --- | --- | --- |
|  | Quintile 1 | Quintile 2 | Quintile 3 | Quintile 4 | Quintile 5 |  |  |
| **Overall plant-based diet index** | |  |  |  |  |  |  |
| Median score (IQR) | 43 (41, 44) | 48 (47, 49) | 51 (50, 52) | 54 (53, 55) | 58 (57, 60) |  |  |
| Minimize model | 0 (ref.) | **-0.32 (-0.49, -0.15)** | **-0.67 (-0.84, -0.50)** | **-0.72 (-0.90, -0.54)** | **-0.96 (-1.13, -0.78)** | <.0001 | **-0.62 (-0.71, -0.52)** |
| Multivariable-adjusted model | 0 (ref.) | -0.13 (-0.28, 0.03) | **-0.36 (-0.53, -0.20)** | **-0.37 (-0.54, -0.20)** | **-0.51 (-0.68, -0.34)** | <.0001 | **-0.34 (-0.44, -0.25)** |
| Multivariable-adjusted without BMI | 0 (ref.) | **-0.21 (-0.38, -0.05)** | **-0.52 (-0.70, -0.35)** | **-0.55 (-0.73, -0.37)** | **-0.79 (-0.97, -0.61)** | <.0001 | **-0.53 (-0.63, -0.43)** |
| **Healthful plant-based diet index** | |  |  |  |  |  |  |
| Median score (IQR) | 47 (44, 48) | 51 (50, 52) | 54 (53, 55) | 57 (56, 58) | 62 (61, 64) |  |  |
| Minimize model | 0 (ref.) | **-0.53 (-0.71, -0.36)** | **-0.67 (-0.84, -0.51)** | **-1.09 (-1.26, -0.93)** | **-1.18 (-1.35, -1.01)** | <.0001 | **-0.73 (-0.82, -0.65)** |
| Multivariable-adjusted model | 0 (ref.) | **-0.33 (-0.49, -0.16)** | **-0.40 (-0.55, -0.24)** | **-0.68 (-0.84, -0.53)** | **-0.71 (-0.87, -0.55)** | <.0001 | **-0.45 (-0.54, -0.36)** |
| Multivariable-adjusted without BMI | 0 (ref.) | **-0.48 (-0.65, -0.30)** | **-0.61 (-0.77, -0.44)** | **-0.99 (-1.16, -0.83)** | **-1.10 (-1.28, -0.93)** | <.0001 | **-0.69 (-0.79, -0.60)** |
| **Unhealthful plant-based diet index** | |  |  |  |  |  |  |
| Median score (IQR) | 48 (46, 49) | 52 (51, 53) | 55 (54, 56) | 58 (57, 59) | 63 (62, 65) |  |  |
| Minimize model | 0 (ref.) | 0.10 (-0.07, 0.27) | **0.25 (0.08, 0.42)** | **0.49 (0.32, 0.65)** | **0.98 (0.81, 1.15)** | <.0001 | **0.56 (0.47, 0.65)** |
| Multivariable-adjusted model | 0 (ref.) | 0.07 (-0.09, 0.23) | **0.23 (0.08, 0.39)** | **0.38 (0.23, 0.53)** | **0.72 (0.56, 0.88)** | <.0001 | **0.41 (0.32, 0.49)** |
| Multivariable-adjusted without BMI | 0 (ref.) | 0.09 (-0.08, 0.25) | **0.22 (0.05, 0.38)** | **0.44 (0.28, 0.60)** | **0.84 (0.67, 1.02)** | <.0001 | **0.49 (0.39, 0.58)** |

Minimize model adjusted for age at the last dietary assessment, age at MRI scan, and sex; Multivariable-adjusted model further adjusted for education, household income, Townsend deprivation index, assessment centers, smoking, alcohol consumption, physical activity, total energy, BMI, NAFLD-PRS, first 10 principal components of ancestry, and genotype measurement batch. We conducted an additional model without BMI as a covariate given that BMI might be in the PDI and NAFLD pathway.

Abbreviations: BMI, body mass index; IQR, interquartile range; MRI, magnetic resonance imaging; NAFLD, non-alcoholic fatty liver disease; PDFF, proton density fat fraction; PRS, polygenic risk score; ref., reference.

**Table S7. Subgroup analysis of the association between overall PDI and the risk of NAFLD by genetic risk**

| **Polygenic risk score group** | No. of cases/ person-years | HR (95% CI) | *P* for  interaction |
| --- | --- | --- | --- |
| **Low PRS** |  |  | 0.16 |
| Tertile 1 | 177/181,441 | 1.00 (ref.) |  |
| Tertile 2 | 135/144,658 | 1.11 (0.89, 1.40) |  |
| Tertile 3 | 101/178,954 | **0.74 (0.58, 0.96)** |  |
| Per 10-point increment |  | 0.84 (0.70, 1.001) |  |
| **Intermediate PRS** |  |  |  |
| Tertile 1 | 210/188,020 | 1.00 (ref.) |  |
| Tertile 2 | 118/145,009 | 0.82 (0.65, 1.02) |  |
| Tertile 3 | 143/184,619 | 0.84 (0.67, 1.05) |  |
| Per 10-point increment |  | **0.82 (0.70, 0.97)** |  |
| **High PRS** |  |  |  |
| Tertile 1 | 272/190,572 | 1.00 (ref.) |  |
| Tertile 2 | 184/148,271 | 1.01 (0.84, 1.22) |  |
| Tertile 3 | 201/188,397 | 0.95 (0.78, 1.14) |  |
| Per 10-point increment |  | 0.97 (0.84, 1.11) |  |

Adjusted for age at the last dietary assessment, sex, education, household income, Townsend deprivation index, assessment centers, smoking, alcohol consumption, physical activity, total energy, BMI, first 10 principal components of ancestry, and genotype measurement batch.

Abbreviations: BMI, body mass index; CI, confidence interval; HR, hazards ratio; NAFLD, non-alcoholic fatty liver disease; PDI, plant-based diet index; PRS, polygenic risk score; ref., reference.

**Table S8. Subgroup analysis of the association between hPDI and the risk of NAFLD by genetic risk**

| **Polygenic risk score group** | No. of cases/ person-years | HR (95% CI) | *P* for  interaction |
| --- | --- | --- | --- |
| **Low PRS** |  |  | 0.03 |
| Tertile 1 | 187/160,034 | 1.00 (ref.) |  |
| Tertile 2 | 118/163,140 | **0.69 (0.55, 0.87)** |  |
| Tertile 3 | 108/181,879 | **0.59 (0.46, 0.77)** |  |
| Per 10-point increment |  | **0.70 (0.59, 0.84)** |  |
| **Intermediate PRS** |  |  |  |
| Tertile 1 | 201/165,729 | 1.00 (ref.) |  |
| Tertile 2 | 143/166,627 | 0.82 (0.66, 1.02) |  |
| Tertile 3 | 127/185,292 | **0.72 (0.57, 0.91)** |  |
| Per 10-point increment |  | **0.79 (0.67, 0.92)** |  |
| **High PRS** |  |  |  |
| Tertile 1 | 262/169,829 | 1.00 (ref.) |  |
| Tertile 2 | 192/168,694 | 0.83 (0.68, 1.003) |  |
| Tertile 3 | 203/188,717 | 0.88 (0.72, 1.07) |  |
| Per 10-point increment |  | 0.88 (0.77, 1.01) |  |

Adjusted for age at the last dietary assessment, sex, education, household income, Townsend deprivation index, assessment centers, smoking, alcohol consumption, physical activity, total energy, BMI, first 10 principal components of ancestry, and genotype measurement batch.

Abbreviations: BMI, body mass index; CI, confidence interval; hPDI, healthful plant-based diet index; HR, hazards ratio; NAFLD, non-alcoholic fatty liver disease; PRS, polygenic risk score; ref., reference.

**Table S9. Subgroup analysis of the association between uPDI and the risk of NAFLD by genetic risk**

| **Polygenic risk score group** | No. of cases/ person-years | HR (95% CI) | *P* for  interaction |
| --- | --- | --- | --- |
| **Low PRS** |  |  | 0.40 |
| Tertile 1 | 105/156,493 | 1.00 (ref.) |  |
| Tertile 2 | 123/163,919 | 1.06 (0.81, 1.37) |  |
| Tertile 3 | 185/184,640 | 1.27 (0.99, 1.63) |  |
| Per 10-point increment |  | **1.25 (1.06, 1.48)** |  |
| **Intermediate PRS** |  |  |  |
| Tertile 1 | 138/159,949 | 1.00 (ref.) |  |
| Tertile 2 | 154/167,730 | 1.05 (0.83, 1.32) |  |
| Tertile 3 | 179/189,968 | 0.99 (0.79, 1.25) |  |
| Per 10-point increment |  | 1.01 (0.86, 1.18) |  |
| **High PRS** |  |  |  |
| Tertile 1 | 174/164,195 | 1.00 (ref.) |  |
| Tertile 2 | 221/170,971 | 1.19 (0.98, 1.46) |  |
| Tertile 3 | 262/192,074 | 1.13 (0.93, 1.37) |  |
| Per 10-point increment |  | **1.17 (1.03, 1.34)** |  |

Adjusted for age at the last dietary assessment, sex, education, household income, Townsend deprivation index, assessment centers, smoking, alcohol consumption, physical activity, total energy, BMI, first 10 principal components of ancestry, and genotype measurement batch.

Abbreviations: BMI, body mass index; CI, confidence interval; HR, hazards ratio; NAFLD, non-alcoholic fatty liver disease; PRS, polygenic risk score; ref., reference; uPDI, unhealthful plant-based diet index.

**Table S10. Hazard ratios (95% confidence intervals) of NAFLD according to sex-specific quintiles of overall plant-based diet index, healthful plant-based diet index, and unhealthful plant-based diet index**

|  | Quintile of dietary score | | | | | *P* for trend | Per 10-point increment |
| --- | --- | --- | --- | --- | --- | --- | --- |
|  | Quintile 1 | Quintile 2 | Quintile 3 | Quintile 4 | Quintile 5 |  |  |
| **Overall plant-based diet index** |  |  |  |  |  |  |  |
| Median score | 43 (41, 45) | 47 (47, 48) | 51 (50, 51) | 53 (53, 54) | 58 (56, 60) |  |  |
| Cases/person-years | 357/298,768 | 354/320,276 | 337/334,481 | 251/286,668 | 242/309,747 |  |  |
| Age and sex-adjusted model | 1.00 (ref.) | 0.91 (0.79, 1.06) | **0.84 (0.73, 0.98)** | **0.73 (0.62, 0.86)** | **0.65 (0.55, 0.77)** | <.0001 | **0.74 (0.68, 0.81)** |
| Multivariable-adjusted model | 1.00 (ref.) | 1.04 (0.89, 1.20) | 1.01 (0.87, 1.18) | 0.92 (0.78, 1.09) | 0.86 (0.73, 1.02) | 0.054 | **0.89 (0.81, 0.97)** |
| Multivariable-adjusted without BMI | 1.00 (ref.) | 0.98 (0.85, 1.14) | 0.94 (0.81, 1.09) | **0.83 (0.70, 0.98)** | **0.75 (0.63, 0.88)** | 0.0002 | **0.81 (0.74, 0.89)** |
| **Healthful plant-based diet index** |  |  |  |  |  |  |  |
| Median score | 48 (46, 49) | 52 (51, 53) | 55 (55, 56) | 59 (58, 59) | 63 (62, 65) |  |  |
| Cases/person-years | 448/330,993 | 300/269,469 | 285/309,190 | 278/350,315 | 230/289,972 |  |  |
| Age and sex-adjusted model | 1.00 (ref.) | **0.82 (0.71, 0.95)** | **0.67 (0.58, 0.78)** | **0.58 (0.50, 0.67)** | **0.58 (0.49, 0.68)** | <.0001 | **0.68 (0.63, 0.74)** |
| Multivariable-adjusted model | 1.00 (ref.) | 0.90 (0.78, 1.04) | **0.79 (0.68, 0.92)** | **0.70 (0.60, 0.82)** | **0.74 (0.63, 0.87)** | <.0001 | **0.80 (0.73, 0.88)** |
| Multivariable-adjusted without BMI | 1.00 (ref.) | **0.83 (0.72, 0.96)** | **0.70 (0.60, 0.81)** | **0.60 (0.51, 0.70)** | **0.59 (0.50, 0.70)** | <.0001 | **0.70 (0.64, 0.76)** |
| **Unhealthful plant-based diet index** |  |  |  |  |  |  |  |
| Median score | 48 (46, 49) | 52 (51, 53) | 55 (55, 56) | 59 (58, 59) | 63 (62, 65) |  |  |
| Cases/person-years | 261/313,738 | 284/305,985 | 322/307,605 | 304/315,979 | 370/306,634 |  |  |
| Age and sex-adjusted model | 1.00 (ref.) | 1.10 (0.93, 1.31) | **1.26 (1.07, 1.49)** | 1.18 (0.999, 1.39) | **1.46 (1.24, 1.72)** | <.0001 | **1.27 (1.16, 1.38)** |
| Multivariable-adjusted model | 1.00 (ref.) | 1.09 (0.92, 1.29) | **1.23 (1.04, 1.45)** | 1.09 (0.92, 1.28) | **1.24 (1.05, 1.46)** | 0.03 | **1.14 (1.05, 1.24)** |
| Multivariable-adjusted without BMI | 1.00 (ref.) | 1.10 (0.93, 1.30) | **1.25 (1.06, 1.47)** | 1.13 (0.96, 1.34) | **1.34 (1.14, 1.58)** | 0.001 | **1.20 (1.10, 1.31)** |

Multivariable-adjusted model further adjusted for education, household income, Townsend deprivation index, assessment centers, smoking, alcohol consumption, physical activity, total energy, BMI, NAFLD-PRS, first 10 principal components of ancestry, and genotype measurement batch. We conducted an additional model without BMI as a covariate given that BMI might be in the PDI and NAFLD pathway.

Abbreviations: BMI, body mass index; NAFLD, non-alcoholic fatty liver disease; PRS, polygenic risk score; ref., reference.

**Table S11. β-coefficient (95% confidence intervals) of MRI-PDFF according to sex-specific quintiles of overall plant-based diet index, healthful plant-based diet index, and unhealthful plant-based diet index**

|  | Quintile of dietary score | | | | | *P* for trend | per 10-point increment |
| --- | --- | --- | --- | --- | --- | --- | --- |
|  | Quintile 1 | Quintile 2 | Quintile 3 | Quintile 4 | Quintile 5 |  |  |
| **Overall plant-based diet index** | |  |  |  |  |  |  |
| Median score (IQR) | 44 (41, 45) | 48 (47, 48) | 51 (50, 51) | 53 (53, 54) | 58 (56, 60) |  |  |
| Minimize model | 0 (ref.) | **-0.25 (-0.42, -0.08)** | **-0.62 (-0.79, -0.45)** | **-0.66 (-0.83, -0.49)** | **-0.92 (-1.09, -0.75)** | <.0001 | **-0.62 (-0.71, -0.52)** |
| Multivariable-adjusted model | 0 (ref.) | **-0.08 (-0.24, 0.08)** | **-0.37 (-0.52, -0.21)** | **-0.34 (-0.50, -0.18)** | **-0.49 (-0.65, -0.33)** | <.0001 | **-0.34 (-0.44, -0.25)** |
| Multivariable-adjusted without BMI | 0 (ref.) | **-0.17 (-0.34, 0.00)** | **-0.49 (-0.65, -0.32)** | **-0.51 (-0.68, -0.34)** | **-0.77 (-0.93, -0.60)** | <.0001 | **-0.53 (-0.63, -0.43)** |
| **Healthful plant-based diet index** | |  |  |  |  |  |  |
| Median score (IQR) | 46 (44, 48) | 51 (50, 52) | 54 (53, 55) | 57 (56, 58) | 62 (61, 65) |  |  |
| Minimize model | 0 (ref.) | **-0.62 (-0.80, -0.45)** | **-0.64 (-0.81, -0.47)** | **-1.12 (-1.29, -0.95)** | **-1.19 (-1.36, -1.02)** | <.0001 | **-0.73 (-0.82, -0.65)** |
| Multivariable-adjusted model | 0 (ref.) | **-0.39 (-0.55, -0.23)** | **-0.32 (-0.49, -0.16)** | **-0.70 (-0.86, -0.54)** | **-0.70 (-0.87, -0.53)** | <.0001 | **-0.45 (-0.54, -0.36)** |
| Multivariable-adjusted without BMI | 0 (ref.) | **-0.58 (-0.75, -0.41)** | **-0.57 (-0.74, -0.40)** | **-1.02 (-1.19, -0.86)** | **-1.11 (-1.29, -0.93)** | <.0001 | **-0.69 (-0.79, -0.60)** |
| **Unhealthful plant-based diet index** | |  |  |  |  |  |  |
| Median score (IQR) | 47 (45, 49) | 52 (51, 53) | 55 (54, 56) | 58 (57, 59) | 63 (62, 65) |  |  |
| Minimize model | 0 (ref.) | 0.13 (-0.05, 0.30) | **0.27 (0.10, 0.44)** | **0.51 (0.34, 0.68)** | **1.00 (0.82, 1.18)** | <.0001 | **0.56 (0.47, 0.65)** |
| Multivariable-adjusted model | 0 (ref.) | 0.08 (-0.08, 0.23) | **0.24 (0.08, 0.40)** | **0.39 (0.23, 0.55)** | **0.73 (0.56, 0.90)** | <.0001 | **0.41 (0.32, 0.49)** |
| Multivariable-adjusted without BMI | 0 (ref.) | 0.12 (-0.05, 0.29) | **0.24 (0.07, 0.41)** | **0.46 (0.30, 0.63)** | **0.87 (0.69, 1.04)** | <.0001 | **0.49 (0.39, 0.58)** |

Minimize model adjusted for age at the last dietary assessment, age at MRI scan, and sex; Multivariable-adjusted model further adjusted for education, household income, Townsend deprivation index, assessment centers, smoking, alcohol consumption, physical activity, total energy, BMI, NAFLD-PRS, first 10 principal components of ancestry, and genotype measurement batch. We conducted an additional model without BMI as a covariate given that BMI might be in the PDI and NAFLD pathway.

Abbreviations: BMI, body mass index; IQR, interquartile range; MRI, magnetic resonance imaging; NAFLD, non-alcoholic fatty liver disease; PDFF, proton density fat fraction; PRS, polygenic risk score; ref., reference.

**Table S12. Subgroup analyses for the associations of PDI, hPDI, and uPDI with the risk of NAFLD per 10-point increment in each index by major confounders**

| Subgroup | No. of cases/ person-years | HR (95% CI) for **PDI** | *P* for  interaction | HR (95% CI) for **hPDI** | *P* for  interaction | HR (95% CI) for **uPDI** | *P* for  interaction |
| --- | --- | --- | --- | --- | --- | --- | --- |
| **Age** |  |  | 0.56 |  | 0.07 |  | 0.60 |
| <60 years | 845/846,276 | **0.87 (0.77, 0.98)** |  | **0.75 (0.67, 0.85)** |  | **1.16 (1.03, 1.29)** |  |
| ≥60 years | 696/703,663 | 0.93 (0.81, 1.06) |  | 0.88 (0.77, 1.01) |  | 1.11 (0.97, 1.26) |  |
| **Obesity** |  |  | 0.07 |  | 0.01 |  | **0.0009** |
| Yes (BMI ≥30 kg/m^2^) | 761/306,082 | 0.91 (0.80, 1.04) |  | **0.88 (0.77, 0.996)** |  | 0.99 (0.88, 1.12) |  |
| No (BMI <30 kg/m^2^) | 780/1,243,857 | **0.84 (0.74, 0.96)** |  | **0.70 (0.61, 0.79)** |  | **1.34 (1.18, 1.51)** |  |
| **Alcohol consumption** |  |  | 0.95 |  | 0.94 |  | 0.75 |
| Below median | 670/690,212 | 0.89 (0.78, 1.03) |  | **0.82 (0.71, 0.94)** |  | 1.16 (1.02, 1.32) |  |
| Above median | 614/683,921 | 0.88 (0.77, 1.02) |  | **0.81 (0.70, 0.94)** |  | 1.13 (0.98, 1.30) |  |
| **Energy intake** |  |  | 0.81 |  | 0.96 |  | 0.54 |
| Below median | 756/776,615 | 0.88 (0.77, 1.01) |  | **0.80 (0.70, 0.91)** |  | **1.17 (1.03, 1.33)** |  |
| Above median | 785/773,325 | 0.90 (0.80, 1.02) |  | **0.81 (0.72, 0.92)** |  | 1.11 (0.99, 1.25) |  |
| **Physical activity** |  |  | 0.50 |  | 0.53 |  | 0.17 |
| <1200 MET-mins/wk | 1,019/888,421 | **0.89 (0.80, 0.997)** |  | **0.81 (0.72, 0.90)** |  | **1.12 (1.01, 1.24)** |  |
| ≥1200 MET-mins/wk | 522/661,518 | 0.87 (0.74, 1.01) |  | **0.78 (0.67, 0.90)** |  | **1.20 (1.04, 1.39)** |  |

Adjusted for age at the last dietary assessment, sex, education, household income, Townsend deprivation index, assessment centers, smoking, alcohol consumption, physical activity, total energy, BMI, NAFLD-PRS, first 10 principal components of ancestry, and genotype measurement batch.

Abbreviations: BMI, body mass index; CI, confidence interval; hPDI, healthful plant-based diet index; HR, hazards ratio; MET, metabolic equivalent of task; NAFLD, non-alcoholic fatty liver disease; PDI, plant-based diet index; PRS, polygenic risk score; uPDI, unhealthful plant-based diet index.

**Table S13. Subgroup analyses for the associations of PDI, hPDI, and uPDI with MRI-PDFF per 10-point increment in each index by major confounders**

| Subgroup | β-coefficient  (95% CI) for **PDI** | *P* for  interaction | β-coefficient  (95% CI) for **hPDI** | *P* for  interaction | β-coefficient  (95% CI) for **uPDI** | *P* for  interaction |
| --- | --- | --- | --- | --- | --- | --- |
| **Age** |  | 0.03 |  | 0.005 |  | 0.15 |
| <60 years | **-0.38 (-0.51, -0.26)** |  | **-0.53 (-0.65, -0.41)** |  | **0.45 (0.33, 0.57)** |  |
| ≥60 years | **-0.28 (-0.42, -0.14)** |  | **-0.38 (-0.51, -0.25)** |  | **0.39 (0.26, 0.52)** |  |
| **Obesity** |  | 0.52 |  | 0.42 |  | 0.78 |
| Yes (BMI ≥30 kg/m^2^) | **-0.41 (-0.75, -0.06)** |  | **-0.46 (-0.79, -0.13)** |  | **0.33 (0.01, 0.65)** |  |
| No (BMI <30 kg/m^2^) | **-0.39 (-0.48, -0.30)** |  | **-0.55 (-0.63, -0.46)** |  | **0.46 (0.37, 0.55)** |  |
| **Alcohol consumption** |  | 0.07 |  | 0.28 |  | 0.67 |
| Below median | **-0.29 (-0.43, -0.15)** |  | **-0.50 (-0.63, -0.37)** |  | **0.46 (0.33, 0.59)** |  |
| Above median | **-0.45 (-0.59, -0.32)** |  | **-0.41 (-0.53, -0.28)** |  | **0.43 (0.30, 0.56)** |  |
| **Energy intake** |  | 0.89 |  | 0.47 |  | 0.44 |
| below median | **-0.35 (-0.48, -0.22)** |  | **-0.49 (-0.61, -0.36)** |  | **0.45 (0.33, 0.57)** |  |
| above median | **-0.33 (-0.46, -0.20)** |  | **-0.40 (-0.53, -0.28)** |  | **0.37 (0.24, 0.50)** |  |
| **Physical activity** |  | 0.45 |  | 0.10 |  | 0.12 |
| <1200 MET-mins/wk | **-0.34 (-0.47, -0.21)** |  | **-0.52 (-0.64, -0.39)** |  | **0.45 (0.33, 0.57)** |  |
| ≥1200 MET-mins/wk | **-0.35 (-0.48, -0.22)** |  | **-0.41 (-0.54, -0.29)** |  | **0.38 (0.26, 0.51)** |  |

Adjusted for age at the last dietary assessment, age at MRI scan, sex, education, household income, Townsend deprivation index, assessment centers, smoking, alcohol consumption, physical activity, total energy, BMI, NAFLD-PRS, first 10 principal components of ancestry, and genotype measurement batch.

Abbreviations: BMI, body mass index; CI, confidence interval; hPDI, healthful plant-based diet index; HR, hazards ratio; MET, metabolic equivalent of task; MRI, magnetic resonance imaging; NAFLD, non-alcoholic fatty liver disease; PDFF, proton density fat fraction; PDI, plant-based diet index; PRS, polygenic risk score; uPDI, unhealthful plant-based diet index.

**Table S14. Sensitivity analyses for associations between plant-based diet indices and NAFLD risk**

|  | Quintile of dietary score | | | | | *P* for trend | Per 10-point increment |
| --- | --- | --- | --- | --- | --- | --- | --- |
|  | Quintile 1 | Quintile 2 | Quintile 3 | Quintile 4 | Quintile 5 |  |  |
| **Overall plant-based diet index** |  |  |  |  |  |  |  |
| Sensitivity analysis 1 | 1.00 (ref.) | 0.94 (0.81, 1.08) | 0.86 (0.73, 1.02) | 0.97 (0.83, 1.12) | **0.79 (0.66, 0.94)** | 0.02 | **0.89 (0.81, 0.98)** |
| Sensitivity analysis 2 | 1.00 (ref.) | 0.93 (0.81, 1.07) | 0.88 (0.74, 1.05) | 0.99 (0.85, 1.15) | **0.80 (0.67, 0.96)** | 0.06 | **0.91 (0.83, 0.998)** |
| Sensitivity analysis 3 | 1.00 (ref.) | 0.91 (0.78, 1.07) | 0.85 (0.70, 1.02) | 0.97 (0.82, 1.14) | **0.80 (0.66, 0.96)** | 0.04 | **0.90 (0.82, 0.99)** |
| Sensitivity analysis 4 | 1.00 (ref.) | 0.91 (0.78, 1.06) | 0.86 (0.71, 1.03) | 0.98 (0.83, 1.14) | **0.81 (0.67, 0.98)** | 0.08 | **0.91 (0.83, 0.996)** |
| Sensitivity analysis 5 | 1.00 (ref.) | 1.02 (0.85, 1.23) | 1.13 (0.92, 1.40) | 0.99 (0.82, 1.21) | 0.80 (0.64, 1.01) | 0.12 | 0.90 (0.80, 1.01) |
| Sensitivity analysis 6 | 1.00 (ref.) | 0.93 (0.80, 1.08) | 0.83 (0.69, 1.002) | 0.93 (0.79, 1.08) | **0.79 (0.65, 0.95)** | 0.02 | **0.87 (0.79, 0.96)** |
| **Healthful plant-based diet index** |  |  |  |  |  |  |  |
| Sensitivity analysis 1 | 1.00 (ref.) | 0.94 (0.81, 1.09) | **0.79 (0.68, 0.93)** | **0.75 (0.64, 0.88)** | **0.77 (0.65, 0.92)** | 0.0001 | **0.82 (0.75, 0.90)** |
| Sensitivity analysis 2 | 1.00 (ref.) | 0.91 (0.78, 1.05) | **0.77 (0.66, 0.90)** | **0.73 (0.62, 0.85)** | **0.76 (0.64, 0.89)** | <0.0001 | **0.81 (0.74, 0.89)** |
| Sensitivity analysis 3 | 1.00 (ref.) | 0.95 (0.81, 1.12) | **0.83 (0.71, 0.98)** | **0.77 (0.65, 0.91)** | **0.74 (0.62, 0.90)** | 0.0001 | **0.83 (0.75, 0.90)** |
| Sensitivity analysis 4 | 1.00 (ref.) | 0.95 (0.81, 1.12) | **0.83 (0.70, 0.98)** | **0.78 (0.65, 0.92)** | **0.75 (0.62, 0.90)** | 0.0003 | **0.83 (0.76, 0.91)** |
| Sensitivity analysis 5 | 1.00 (ref.) | 0.85 (0.71, 1.02) | **0.65 (0.53, 0.79)** | **0.69 (0.55, 0.86)** | **0.75 (0.61, 0.92)** | 0.0005 | **0.81 (0.72, 0.91)** |
| Sensitivity analysis 6 | 1.00 (ref.) | 0.91 (0.78, 1.07) | **0.80 (0.68, 0.94)** | **0.73 (0.62, 0.86)** | **0.74 (0.62, 0.88)** | <0.0001 | **0.80 (0.73, 0.88)** |
| **Unhealthful plant-based diet index** |  |  |  |  |  |  |  |
| Sensitivity analysis 1 | 1.00 (ref.) | 1.10 (0.92, 1.31) | **1.21 (1.03, 1.43)** | 1.10 (0.93, 1.29) | **1.21 (1.03, 1.43)** | 0.04 | **1.12 (1.03, 1.22)** |
| Sensitivity analysis 2 | 1.00 (ref.) | 1.06 (0.89, 1.27) | **1.22 (1.03, 1.44)** | 1.09 (0.93, 1.28) | **1.22 (1.04, 1.44)** | 0.02 | **1.13 (1.04, 1.23)** |
| Sensitivity analysis 3 | 1.00 (ref.) | 1.04 (0.86, 1.26) | 1.19 (0.997, 1.42) | 1.06 (0.89, 1.27) | 1.15 (0.96, 1.37) | 0.06 | **1.11 (1.02, 1.21)** |
| Sensitivity analysis 4 | 1.00 (ref.) | 1.04 (0.86, 1.27) | 1.20 (1.01, 1.44) | 1.08 (0.91, 1.29) | 1.15 (0.96, 1.37) | 0.06 | **1.11 (1.02, 1.21)** |
| Sensitivity analysis 5 | 1.00 (ref.) | 1.02 (0.82, 1.27) | 1.03 (0.84, 1.27) | **1.22 (1.002, 1.48)** | 1.17 (0.95, 1.44) | 0.04 | **1.16 (1.04, 1.30)** |
| Sensitivity analysis 6 | 1.00 (ref.) | 1.05 (0.87, 1.26) | **1.19 (1.002, 1.42)** | 1.07 (0.90, 1.26) | 1.17 (0.99, 1.39) | 0.10 | **1.11 (1.02, 1.22)** |

Sensitivity analysis 1: further adjusted for diagnosed depression, dyslipidemia, hypertension, and diabetes at the last dietary assessment completed;

Sensitivity analysis 2: further adjusted for baseline albumin, alanine aminotransferase, and gamma-glutamyltransferase;

Sensitivity analysis 3: further adjusted for glucose, glycated haemoglobin, and triglyceride;

Sensitivity analysis 4: further adjusted for waist circumference;

Sensitivity analysis 5: excluded participants with less than twice dietary assessment, n=105,553;

Sensitivity analysis 6: excluded participants with less than 2 years of follow-up, n=158,346.

Abbreviations: BMI, body mass index; NAFLD, non-alcoholic fatty liver disease; ref., reference.

**Table S15. Mediating effect of BMI on associations between** **plant-based diet indices and NAFLD risk**

|  | Direct effect, HR (95% CI) | Total effect, HR (95% CI) | Proportion of mediation | *P*-value |
| --- | --- | --- | --- | --- |
| **Overall plant-based diet index** | 0.97 (0.95, 0.99) | 0.99 (0.96, 1.01) | 51.8% (16.6%, 85.3%) | <.0001 |
| **Healthful plant-based diet index** | 0.96 (0.94, 0.99) | 0.98 (0.96, 1.00) | 47.0% (21.6%, 74.0%) | <.0001 |
| **Unhealthful plant-based diet index** | 1.02 (0.99, 1.04) | 1.01 (0.99, 1.03) | 46.5% (5.7%, 92.5%) | <.0001 |

Adjusted for age at the last dietary assessment, sex, education, household income, Townsend deprivation index, assessment centers, smoking, alcohol consumption, physical activity, total energy, NAFLD-PRS, first 10 principal components of ancestry, and genotype measurement batch.

Abbreviations: BMI, body mass index; CI, confidence interval; HR, hazards ratio; NAFLD, non-alcoholic fatty liver disease; PRS, polygenic risk score.

**Table S16. Hazard ratio (95%** **confidence intervals) for NAFLD according to modified plant-based diet indices (per 10-point increment) with additional adjustment for** **the excluded food group (servings/day)**

|  | **Overall plant-based diet** | **Excluded food group** |
| --- | --- | --- |
| Whole grains | **0.90 (0.82, 0.99)** | 0.97 (0.93, 1.01) |
| Fruits | **0.90 (0.81, 0.99)** | 0.99 (0.97, 1.02) |
| Vegetables | **0.90 (0.81, 0.99)** | 0.99 (0.97, 1.02) |
| Nuts | **0.90 (0.82, 0.99)** | **0.86 (0.74, 0.99)** |
| Legumes | **0.88 (0.80, 0.97)** | 0.99 (0.88, 1.10) |
| Tea and coffee | 0.93 (0.84, 1.02) | **0.94 (0.92, 0.97)** |
| Refined grains | **0.90 (0.82, 0.99)** | 1.01 (0.96, 1.05) |
| Potatoes | **0.86 (0.78, 0.95)** | 1.05 (0.97, 1.13) |
| Sugar-sweetened beverages | **0.85 (0.77, 0.93)** | **1.13 (1.08, 1.18)** |
| Fruit juices | **0.87 (0.80, 0.96)** | 1.07 (0.98, 1.16) |
| Sweets and desserts | **0.90 (0.82, 0.99)** | 0.97 (0.94, 1.01) |
| Animal fat | **0.89 (0.81, 0.98)** | 1.01 (0.97, 1.04) |
| Dairy | **0.87 (0.79, 0.95)** | 0.98 (0.93, 1.04) |
| Eggs | **0.90 (0.81, 0.99)** | 1.06 (0.97, 1.15) |
| Fish and sea foods | **0.91 (0.83, 0.99)** | **1.13 (1.03, 1.24)** |
| Meat | **0.90 (0.81, 0.98)** | 1.02 (0.98, 1.07) |
| Miscellaneous animal-based foods | **0.88 (0.80, 0.97)** | 0.95 (0.83, 1.08) |
|  | **Healthful plant-based diet** | **Excluded food group** |
| Whole grains | **0.79 (0.72, 0.87)** | 0.99 (0.95, 1.03) |
| Fruits | **0.78 (0.71, 0.87)** | 1.01 (0.98, 1.04) |
| Vegetables | **0.79 (0.72, 0.87)** | 1.00 (0.98, 1.02) |
| Nuts | **0.81 (0.74, 0.89)** | 0.88 (0.76, 1.02) |
| Legumes | **0.78 (0.71, 0.86)** | 1.01 (0.90, 1.12) |
| Tea and coffee | **0.84 (0.76, 0.92)** | **0.95 (0.92, 0.97)** |
| Refined grains | **0.78 (0.71, 0.85)** | 1.01 (0.97, 1.06) |
| Potatoes | **0.81 (0.74, 0.89)** | 1.07 (0.99, 1.15) |
| Sugar-sweetened beverages | **0.85 (0.78, 0.94)** | **1.13 (1.08, 1.18)** |
| Fruit juices | **0.80 (0.73, 0.87)** | 1.08 (0.99, 1.17) |
| Sweets and desserts | **0.77 (0.71, 0.85)** | 0.98 (0.94, 1.02) |
| Animal fat | **0.80 (0.73, 0.88)** | 1.01 (0.97, 1.04) |
| Dairy | **0.79 (0.72, 0.86)** | 0.996 (0.94, 1.06) |
| Eggs | **0.80 (0.73, 0.88)** | 1.08 (0.99, 1.18) |
| Fish and sea foods | **0.81 (0.74, 0.88)** | **1.15 (1.05, 1.27)** |
| Meat | **0.80 (0.73, 0.88)** | 1.03 (0.98, 1.08) |
| Miscellaneous animal-based foods | **0.80 (0.73, 0.87)** | 0.97 (0.85, 1.11) |
|  | **Unhealthful plant-based diet** | **Excluded food group** |
| Whole grains | **1.13 (1.03, 1.24)** | 0.98 (0.94, 1.02) |
| Fruits | **1.14 (1.03, 1.25)** | 0.996 (0.97, 1.03) |
| Vegetables | **1.13 (1.03, 1.24)** | 0.995 (0.97, 1.02) |
| Nuts | **1.12 (1.03, 1.23)** | **0.86 (0.74, 0.99)** |
| Legumes | **1.15 (1.05, 1.26)** | 0.98 (0.88, 1.09) |
| Tea and coffee | 1.08 (0.98, 1.18) | **0.95 (0.92, 0.97)** |
| Refined grains | **1.17 (1.07, 1.28)** | 1.01 (0.97, 1.05) |
| Potatoes | **1.12 (1.03, 1.23)** | 1.05 (0.97, 1.13) |
| Sugar-sweetened beverages | 1.06 (0.97, 1.16) | **1.14 (1.09, 1.19)** |
| Fruit juices | **1.14 (1.04, 1.24)** | 1.07 (0.99, 1.16) |
| Sweets and desserts | **1.20 (1.10, 1.32)** | 0.97 (0.93, 1.002) |
| Animal fat | **1.16 (1.06, 1.26)** | 1.004 (0.97, 1.04) |
| Dairy | **1.13 (1.03, 1.23)** | 0.98 (0.92, 1.04) |
| Eggs | **1.17 (1.07, 1.28)** | 1.07 (0.98, 1.17) |
| Fish and sea foods | **1.17 (1.07, 1.28)** | **1.11 (1.01, 1.22)** |
| Meat | **1.15 (1.05, 1.25)** | 1.02 (0.97, 1.07) |
| Miscellaneous animal-based foods | **1.14 (1.04, 1.24)** | 0.94 (0.83, 1.08) |

Adjusted for age at the last dietary assessment, sex, education, household income, Townsend deprivation index, assessment centers, smoking, alcohol consumption, physical activity, total energy, BMI, the excluded food group intake, NAFLD-PRS, first 10 principal components of ancestry, and genotype measurement batch.

Abbreviations: BMI, body mass index; NAFLD, non-alcoholic fatty liver disease; PRS, polygenic risk score.
